# Multi-region and single-cell sequencing reveal variable genomic heterogeneity in rectal cancer

**DOI:** 10.1186/s12885-017-3777-4

**Published:** 2017-11-23

**Authors:** Mingshan Liu, Yang Liu, Jiabo Di, Zhe Su, Hong Yang, Beihai Jiang, Zaozao Wang, Meng Zhuang, Fan Bai, Xiangqian Su

**Affiliations:** 1 Biodynamics Optical Imaging Center (BIOPIC), School of Life Sciences, Peking University, No. 5 Yiheyuan Road, Haidian District, Beijing, 100871 China; 2 Key Laboratory of Carcinogenesis and Translational Research (Ministry of Education), Department of Gastrointestinal Surgery IV, Peking University Cancer Hospital & Institute, 52 Fucheng Road, Haidian District, Beijing, 100142 China

**Keywords:** Rectal cancer, Single-cell whole-genome sequencing, Multi-region whole-exome sequencing, Somatic copy number alterations, Intratumor heterogeneity

## Abstract

**Background:**

Colorectal cancer is a heterogeneous group of malignancies with complex molecular subtypes. While colon cancer has been widely investigated, studies on rectal cancer are very limited. Here, we performed multi-region whole-exome sequencing and single-cell whole-genome sequencing to examine the genomic intratumor heterogeneity (ITH) of rectal tumors.

**Methods:**

We sequenced nine tumor regions and 88 single cells from two rectal cancer patients with tumors of the same molecular classification and characterized their mutation profiles and somatic copy number alterations (SCNAs) at the multi-region and the single-cell levels.

**Results:**

A variable extent of genomic heterogeneity was observed between the two patients, and the degree of ITH increased when analyzed on the single-cell level. We found that major SCNAs were early events in cancer development and inherited steadily. Single-cell sequencing revealed mutations and SCNAs which were hidden in bulk sequencing. In summary, we studied the ITH of rectal cancer at regional and single-cell resolution and demonstrated that variable heterogeneity existed in two patients. The mutational scenarios and SCNA profiles of two patients with treatment naïve from the same molecular subtype are quite different.

**Conclusions:**

Our results suggest each tumor possesses its own architecture, which may result in different diagnosis, prognosis, and drug responses. Remarkable ITH exists in the two patients we have studied, providing a preliminary impression of ITH in rectal cancer.

**Electronic supplementary material:**

The online version of this article (10.1186/s12885-017-3777-4) contains supplementary material, which is available to authorized users.

## Background

Colorectal cancer is highly heterogeneous, and its pathogenesis and molecular classification have been widely investigated [1, 2]. In fact, colon and rectal cancers not only have different clinicopathological features, but also undergo different molecular paths of tumorigenesis [3]. Tumor heterogeneity, a notable feature of cancer, has recently been studied in breast cancer [4], esophageal cancer [5], renal cancer [6, 7] and lung cancer [8, 9] through multi-region sequencing of tumor masses. Intratumor heterogeneity (ITH) and branched evolution were commonly observed, and the complexity of the tumor tissue composition was beyond expectation. However, tumor heterogeneity of colorectal cancer, especially rectal cancer, was less investigated.

ITH can be assessed by single-cell sequencing, as recent progress in single-cell genome sequencing has allowed quantitative characterization of both single nucleotide variations (SNVs) and somatic copy number alterations (SCNAs) in individual tumor cells. For instance, single-cell sequencing of individual circulating tumor cells (CTCs) revealed reproducible SCNA patterns in CTCs from the same patient and identified pertinent cancer mutations [10]. Single-cell sequencing of a large number of breast tumor cells [11–13] revealed punctuated evolution of SCNAs during tumor development. In addition, single-cell exome sequencing analysis of a case of colon cancer revealed a biclonal tumor origin and proved low-prevalence mutations could also play a role in tumorigenesis [14]. Nevertheless, the ITH of rectal cancer has not been well studied by single-cell sequencing.

In the current study, we performed multi-region whole-exome sequencing (WES) and single-cell whole-genome sequencing (WGS) to evaluate the ITH of two rectal tumors. The SCNAs and mutations were exquisitely identified from multi-region to single-cell level. We found that the extent of ITH in the two patients was variable, and the degree of heterogeneity increased when analyzed on the single-cell level.

## Methods

### Sample collection and single cell preparation

We obtained two fresh primary rectal tumors from patients who underwent primary tumor resection at the Department of Gastrointestinal Surgery IV, Peking University Cancer Hospital & Institute. None of them received radiotherapy or chemotherapy before surgery. The clinicopathological characteristics of the two patients are listed in Additional file 1: Table S1. Sections were collected from different regions of tumors immediately after surgical removal. To obtain single-cell suspensions, each region was washed, minced with sterile blades into small pieces, and dissociated by incubation in DMEM containing collagenase type IA (50 μg/mL; Sigma-Aldrich Co. LLC, US), hyaluronidase (20 μg/μL; Sigma-Aldrich Co. LLC, US), and antibiotics/antimyotics for 1 h at 37 °C. After digestion, cells were filtered through a 70 μm cell strainer (BD Falcon™, US), and erythrocytes were removed by treatment with NH_4_Cl/EDTA. Cells were then cryopreserved in liquid nitrogen. Peripheral blood from each patient was collected and stored at −20 °C.

### The fluorescent activated cell sorting (FACS) and single-cell isolation

To isolate single tumor cells, cryopreserved cells were thawed and stained with combinations of the following reagents: anti-EpCAM Alexa Fluor® 488 (eBioscience, US), and lineage-specific antibodies, including anti-CD45-PE (BD Pharmingen™, US), anti-CD235a-PE (BD Pharmingen™, US), anti-CD140b-PE (BD Pharmingen™, US), and anti-CD31-PE (BD Pharmingen™, US). To discriminate viable cells, 7-Amino-Actinomycin D (7-AAD, BD Pharmingen™, US) was labeled 5–10 min before sorting. Single tumor cells were sorted based on 7-AAD^−^lineage^−^EpCAM^high^ by BD FACS Aria III (BD Biosciences, US). Individual tumor cells were verified under the fluorescence microscopy (Nikon Eclipse Ti, Japan) and separated by mouth pipetting. Isolated single cells were then lysed.

### Whole-exome library preparation and sequencing

We used the QIAamp Micro DNA kit (QIAGEN, US) to extract genomic DNA from the single-cell suspension derived from sections and matched blood, and the concentrations were measured by Qubit 2.0 fluorometer (Invitrogen, US). Total gDNA (~600 ng) was sheared into fragments (~180–280 bp) by the Covaris system (Covaris, US). Libraries were generated using the Agilent SureSelect Human All Exon V6 kit (Agilent Technologies, US) following the manufacturer’s recommendations, and index codes were added to each sample. The products were sequenced with Illumina Hiseq4000 2 × 150-bp PE reads at ~100× depth.

### Whole-genome library preparation and sequencing

After lysis, single cells were amplified by the multiple annealing and looping-based amplification cycles (MALBAC) method [15]. The cells passed the quantitative PCR (qPCR) quality control [10] were used for next-generation sequencing (Bio-Rad, US). DNA (~600 ng) from each single cell and gDNA (~500 ng) from tumor tissue was sheared into ~300 bp fragments by the Covaris system (Covaris, US), and the indexed libraries were prepared with the NEBNext Ultra DNA Library Prep Kit for Illumina (New England Biolabs, US). The products were then sequenced with Illumina HiseqXTen 2 × 150-bp PE reads at ~0.3× depth.

### Analysis of WES data

The reads were aligned to the human reference genome (hg19, USCC) with the Burrows-Wheeler Aligner [16]. The aligned BAM files were sorted and merged with Samtools 0.1.19 [17]. First, we applied two software, the Genome Analysis Toolkit (GATK 1.6) [18] and multiSNV [19], to identify mutations in multi-region WES. The INDELs and SNVs were identified with GATK 1.6 [18] based on dbSNP 135 (www.ncbi.nlm.nih.gov/projects/SNP/), and the duplicates were removed with Picard-tools 1.76 (http://Picard.Sourceforge.net). The functional effect of variants was annotated using SNPEFF3.0 [20]. Then, the SNVs and INDELs (insertion and deletion) were filtered out based on previous criteria [21] using the Catalog of Somatic Mutations in Cancer (COSMIC) database v61. We manually filtered out tumor mutations with base quality of lower than 30 and distance between two mutations of lower than 15 bp. Germline mutations were removed by comparing the tumor data to matched blood data. Next, we input the aligned BAM files into multiSNV [19] to call the SNVs. Germline SNPs were removed by comparing the tumor data to matched blood data. After that, low quality SNPs were filtered and the functional effect of variants was annotated using SNPEFF3.0 [20]. Shared SNVs of each region by the two software were used for subsequent analysis. Additionally, to reduce the false negative rate, we had manually assessed the SNVs which had low allelic frequency in samples. Some SNVs existed in two or more samples of one patient, but were detected by either software in only one sample. Then we would screened manually in these SNVs, of which if variant allelic frequency (VAF) in samples was more than 0.2 we would put them back into our SNV list. Eventually, we added the INDELs identified by GATK into the shared SNV list to get the final mutations for further analysis.

Phylogenetic trees were constructed by MEGA5 with maximum likelihood method [22], and potential driver mutations were labelled on branches with Adobe Illustrator. The purities and SCNA profiles of multiple tumor regions from one patient were estimated with the Sequenza R package 2.1.1 [23].

### The SCNA profiles of the tumor regions

The libraries of tumor regions and match blood constructed with gDNA were performed WGS. The clean data was aligned to human reference genome (hg19, UCSC) with the Burrows-Wheeler Aligner [16]. After that, we sorted and merged each sample with Samtools 0.1.19 [17]. To visualize the SCNA profiles of WGS, we sorted the whole genome into 500Kb bins (on average), and then used matched blood as control to remove noises. Finally, the depth of each bin of tumor regions was plotted along the order of the chromosomes.

### The single-cell SCNA profiling

The single-cell SCNA profiles were identified using previously described methods [10, 15]. The reads were aligned to human reference genome (hg19, UCSC) with the Burrows-Wheeler Aligner [16] and then sorted and merged with Samtools 0.1.19 [17]. The whole genome was sorted into 500Kb bins (on average), and the depth of each bin was determined by the hidden Markov model normalized with the method control [10].

### Single-cell WGS analyses

The median of the absolute values of all pairwise differences (MAPD) was used to assess the quality of the single-cell data [24]. The MAPD scores of the 88 cells were less than 0.25, and all of them passed the quality control. The clustered heat map of the large-scale copy number profiles was generated by the Euclidean distance and ward.D method and visualized by the heatmap.2 function in the gplots package. The principle component analysis (PCA) was performed with the prcomp function in the stats package. Partition around medoids (PAM) clustering was performed using the pamk function in the fpc package. The consensus copy number profiles of multiple regions were inferred from single tumor cells based on the median value of each bin.

### Identification of subclonal SCNAs

The subclonal SCNAs of single cells were identified by PCA using the FactoMineR package based on the depth of each bin (each patient had 6037 bins at 500Kb) and were visualized with the gplots package. We integrated the bins of single tumor cells from each patient into one matrix and filtered out the bins with all elements equal to zero. Each included bin had at least three elements greater than zero. Then, we set the variance of each bin to greater than 0.5 to obtain subclonal SCNAs with high disparities. There were 116 and 1637 bins containing subclonal SCNAs collected from PC1 to PC6 for patients 1 and 2, respectively. After that, we manually selected subclonal SCNAs larger than 1.5 Mb (63 and 806 bins for patients 1 and 2, respectively), and visualized the results with clustered heat maps.

### Single-cell mutation validation

The mutations identified in the multi-region WES were validated in single cells by Sanger sequencing (Ruibiotech, China) using 20 ng of the MALBAC products as DNA templates. The PCR was performed with OneTaq Hot Start Quick-Load 2× Master Mix (New England Biolabs, US). The thermal profile was 94 °C for 60 s; 35 cycles of 94 °C for 25 s, 58 °C for 30 s, and 68 °C 40 s; and 68 °C for 5 mins. The primers used are listed in Additional file 1: Table S2.

We used ploidy status and ubiquitous mutations to distinguish somatic diploid cells and tumor cells. We used five or six nonsynonymous ubiquitous mutations which were identified in multi-region WES as candidate mutations to exclude somatic diploid cells (Additional file 1: Table S3). A single cell was considered to be somatic diploid cells if the candidate mutations were validated as wildtype by Sanger sequencing, while tumor cells had SCNAs and mutations. Owing to allelic dropout and imbalanced single-cell amplification, some mutations were undetectable in single cells, but were validated in gDNA of the tumor. As shown in Table S3, the candidate mutations were all validated in the gDNA of the two tumors, but sporadically identified in single cells. There were 15 diploid cells excluded in patient 1, of which two cells (B1 and C8) containing more than three mutations were excluded in the later analysis, owing to the possibility that they were a mixture of one diploid cell and debris of tumor cells. The number of diploid cells in patient 2 was 13, and none of the six candidate mutations were validated in them. In total, 26 cells (13 from patient 1 and 13 from patient 2) were confirmed to be somatic diploid cells, and two cells (B1 and C8 of patient 1) seemed to be mixtures, which were all excluded in further analysis of tumor cells.

Considering the phylogenetic trees, putative driver mutations in the COSMIC database, disease-associated genes identified by DAVID [25, 26] and possible driver mutations in cancer genome landscape [27], we selected 14 nonsynonymous mutations for each patient and validated the presence of these WES identified mutations in single tumor cells with SCNAs. The single cells with SCNAs were confirmed to be tumor cells if at least four mutations were present.

## Results

### Multi-region WES revealed variable genomic heterogeneity

**Fig. 1:**
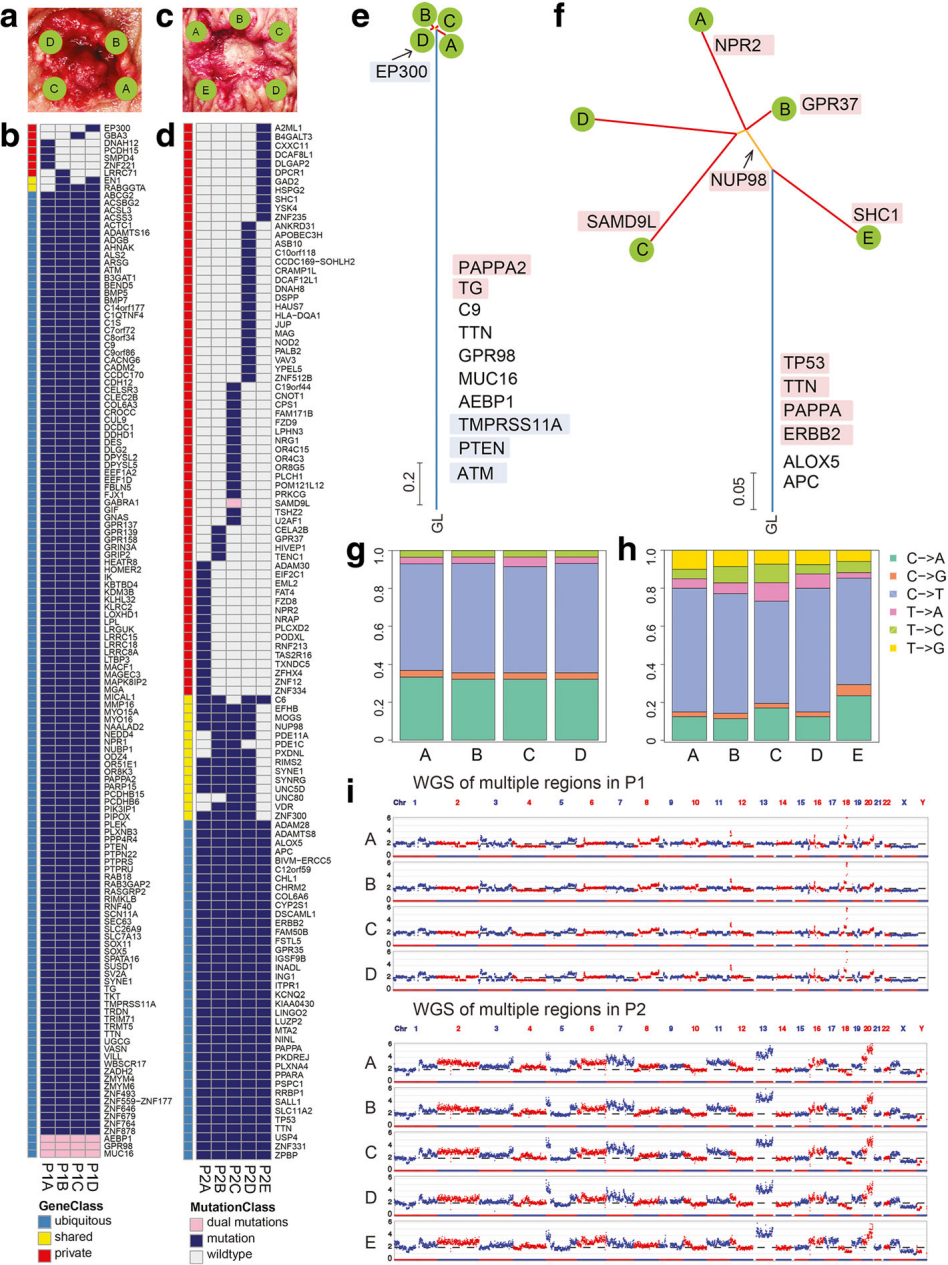
Multi-region WES revealed variable genomic heterogeneity in two rectal tumors. **a** The multiple regions of patient 1 divided by physical distance. **b** The distribution of nonsynonymous mutations in multiple regions of patient 1. The blue and the grey in heat map presented the mutations and the absences, respectively. The pink in heat map means this gene had two separate independent mutations. The color bars next to the heat map indicate classification of mutations according to whether they are ubiquitous, shared by some tumor regions but not all, or unique to the region (private). **c** The multiple regions of patient 2 divided by physical distance. **d** The distribution of nonsynonymous mutations in multiple regions of patient 2. **e** The phylogenetic tree of patient 1 deduced from multi-region WES. The blue trunk, yellow branches and red leaves represented the clonal, the subclonal and the private mutations, respectively. The red, the white and the blue background of mutations meant the gain (>2 N), normal (~2 N) and loss (<2 N) of copy number, respectively. The distance of the branch was based on similar probability between samples. **f** The phylogenetic tree of patient 2 deduced from multi-region WES. **g** The mutation spectrum of multiple regions in patient 1. **h** The mutation spectrum of multiple regions in patient 2. **i** The copy number profiles of multiple regions and blood in patient 1 and patient 2. The SCNAs of genomic DNA from multiple regions (blue) and matched blood (red) detected by whole-genome sequencing was visualized by Circos. P1: patient1; P2: patient 2

To depict the genomic heterogeneity of rectal cancer, multi-region WES was performed to determine the mutation distribution and SCNAs profiles in the two rectal primary tumors. The two fresh primary rectal tumors were of the same molecular subtype [28], which was microsatellite stable, chromosomal instable (referring to SCNAs here), and/or mutant TP53 with wildtype KRAS and PIK3CA (Additional file 1: Table S1). To obtain mutational profiles, we carried out WES on multiple regions and matched blood (germline comparator) at ~100× depth (Additional file 1: Table S4). For patient 1, four regions (A to D) were sequenced (Fig. 1a), and 141 nonsynonymous mutations involving 138 genes were detected (Fig. 1b, Additional file 1: Table S5). In the five regions (A to E) of patient 2 (Fig. 1c), 119 nonsynonymous mutations involving 117 genes were identified (Fig. 1d, Additional file 1: Table S5). The mutations were categorized as ‘ubiquitous’, which were mutations shared by all regions of the tumor, ‘shared’, which were shared by more than one region but not all regions, and ‘private’, which were specific to a single region. According to the phylogenetic trees which delineated the tumor evolutionary patterns (Fig. 1e and f) and the heat maps of nonsynonymous mutations (Fig. 1b and d), analysis of the regional distribution of nonsynonymous mutations revealed more ITH in patient 2 than that in patient 1. The observation that the mutational heterogeneity of patient 2 was more extensive than that of patient 1 might be due to the fact that the tumor from patient 2 was larger in size and later in stage (Additional file 1: Table S1), implying that a longer disease progression might foster tumor heterogeneity.

As the mutation spectrums showed, C > T transitions were prominent in both patients (Fig. 1g and h). There was no significant difference in the mutation spectrum among the tumor regions of patient 1 (χ-squared test, *p* > 0.05). T > A transversions were detected in patient 2 among the shared and private mutations, especially in region C (Fisher’s exact test, *p* < 0.05), suggesting that different tumor microenvironment might bring about the differences in mutational profiles [29].

We combined VAF, copy number, and the purity of tumor tissue to analyze the cancer cell fraction of each region as a means to discriminate mutational heterogeneity of each region [30]. As shown in Additional file 1: Fig. S1 and Fig. S2, patient 2 had much more mutations on axes (marked by green and blue) than patient 1, which were referred to region-specific subclones. Therefore, the multiple regions in patient 2 were more heterogeneous than those of patient 2. Moreover, the mutational scenarios of the two patients were quite different. In patient 1, mutations in *ATM* and *GNAS*, as well as a deletion in the tumor suppressor gene *PTEN*, likely led to tumorigenesis since they are potential cancer driver genes [2, 27]. In patient 2, mutations in *TP53*, *ERBB2* and *APC*, which were frequently mutated in colorectal tumors and involved in the WNT/β-catenin signalling pathway [31], might play important roles in tumorigenesis and could be possible drug targets [32, 33].

Gene mutations are associated with chromosomal instability, a consequence of which is SCNAs [34], and the interactions of these two events facilitate tumor progression. We performed WGS on multiple tumor regions and matched blood at ~0.3× depth to depict SCNA profiles of each tumor region. The SCNA profiles of the tumor regions for each patient were found to be very similar (mean Pearson correlation coefficient of patient 1 and patient 2 was 0.9713 and 0.9822, respectively) and highly reproducible (Fig. 1i). The genomes of both patients had gains at chr20q and losses at chr18q, which were accordant with the previously reported frequent copy number changes in colorectal cancer [35]. In addition, we observed common SCNA gains in these two patients at chr1q21-23, chr3q27-28, chr5q32-35, chr6p21, chr8q23-24, chr16p11 and chr17q25, as well as SCNA losses at chr1p22 and chr9q12. Patient 1 had losses at chrX, while patient 2 had gains at chrX. Given that the WGS was performed at a low depth of coverage, to improve the resolution of more focal events, we analyzed SCNA profiles with the WES data eliminating the contamination caused by diploid cells by using Sequenza. The SCNA profiles of the tumor regions in patient 1 also seemed to be similar, while those of certain regions in patient 2 were obviously distinguishable at chr3q and chr8p among the five regions (Additional file 1: Fig. S3). Collectively, these data indicate that the SCNA profiles of the tumor cells in patient 2 were more heterogeneous, and multi-region WES was not sufficient to fully represent the full scenarios of the SCNA profiles.

### Single-cell sequencing showed SCNA-based subpopulations

**Fig. 2:**
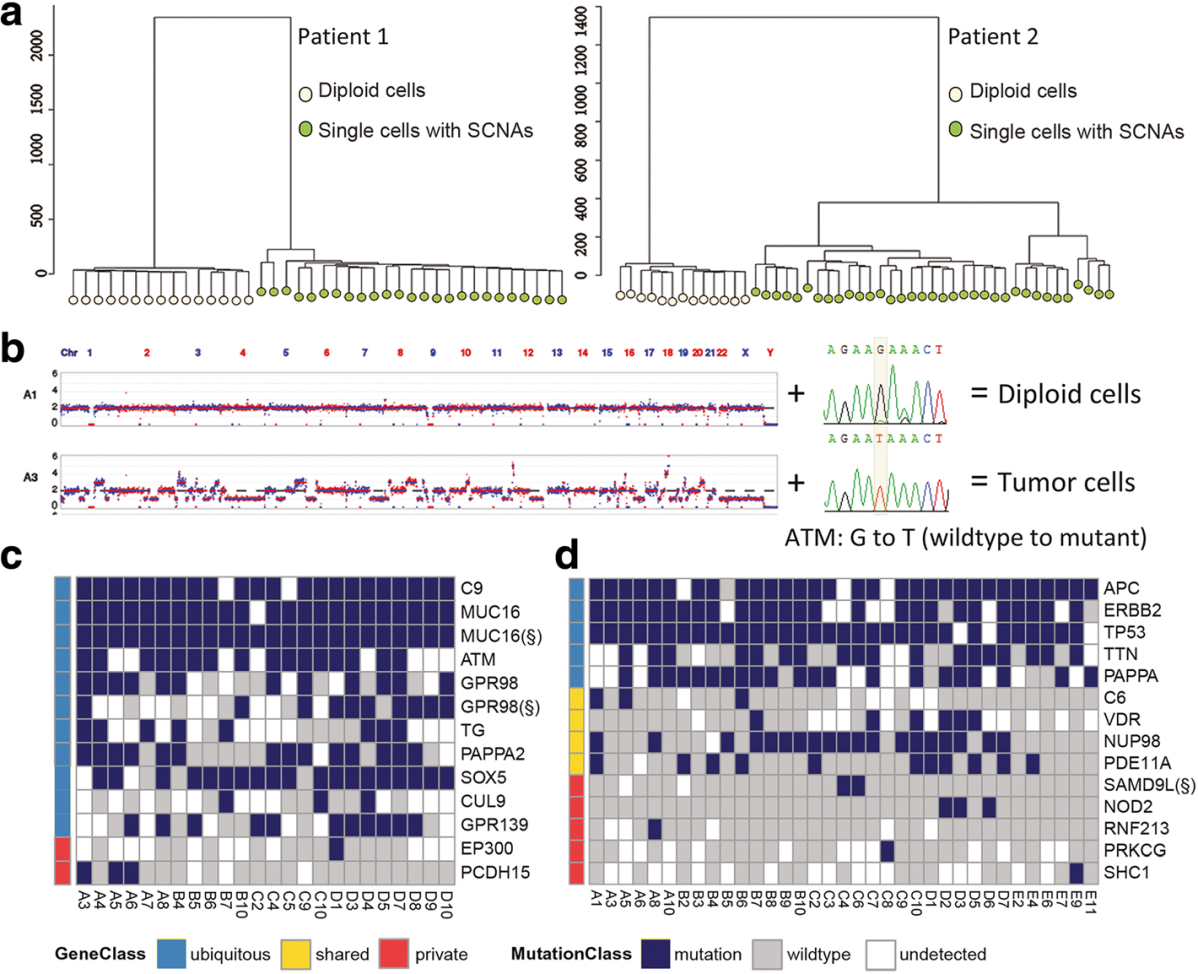
Single-cell sequencing showed SCNA-based subpopulations within two rectal tumors. **a** Cluster analysis of single cells of each patient based on copy number profiles. The cluster was constructed by Euclidean distance and ward.D method. The yellow and the green represented diploid cells and tumor cells with SCNAs, respectively. **b** The procedure to distinguish between diploid normal and tumor cells. Diploid cells without mutations were considered to be normal cells, while cells with both SCNAs and mutations were considered as tumor cells. **c** The mutations validated in single tumor cells of patient 1 by Sanger sequencing. The blue, the grey and the white presented the mutations, the absence of mutations and the undetected by PCR, respectively. (§) represented this gene had two separate independent mutations (**d**) The mutations validated in single tumor cells of patient 2 by Sanger sequencing. *SAMD9L*(§) had two base substitution TC to AA at chr7: 92,763,288-92,763,289

We performed single-cell WGS to access the ITH of each region at the single-cell level. Tumor cells were sorted by FACS based on the 7-AAD^−^Lineage^−^EpCAM^high^ biomarker combination [36] and then single cells were picked up by micropipetting under microscope. Genomic DNA of each cell was amplified using MALBAC [15], an outstanding whole genome amplification method that allows accurate detection of SCNAs and mutations from single cells [37, 38]. The SCNA profile of each cell was plotted using previously established protocols [10, 15]. In total, 40 single cells of patient 1 (ten single cells for each region) and 48 single cells of patient 2 (eight single cells for region D and ten single cells for the other regions) passed the quality control and were subjected to single-cell WGS. Hierarchical clustering showed that the single cells of each patient were divided into two subpopulations, diploid cells and cells with SCNAs (Fig. 2a). PAM clustering [39] was applied to quantify the number of clusters, which also supported the results (Additional file 1: Fig. S4).

We then analyzed the presence of the WES detected mutations in each single cell, and the procedure of normal and tumor cell validation was shown in Fig. 2b. There was a possibility that even after the 7-AAD^−^Lineage^−^EpCAM^high^ enrichment, there were still a few normal stroma cells mixed in the tumor cell population, and these diploid cells were ruled out in the validation procedure (Additional file 1: Table S3). In single tumor cells with SCNAs, we selected a set of mutations identified by multi-region WES and assessed their presence by targeted PCR and Sanger sequencing to exclude the calling of false-positive SNVs inherited from single-cell whole-genome amplification. After validating mutations by Sanger sequencing, we were able to confirm 24 out of 40 cells from patient 1 (Fig. 2c, six for region A, five for region B, five for region C, and eight for region D, Additional file 1: Table S6) and 35 out of 48 cells from patient 2 (Fig. 2d, six for region A, nine for region B, eight for region C, six for region D, and six for region E, Additional file 1: Table S6) as tumor cells with genomes that acquired SCNAs and possessed cancer-associated mutations simultaneously. Of special note, the mutation in PDE11A gene was ‘shared’ mutation by regions B, C and D in patient 2 (Fig. 1b). However, we found that it also existed in a single cells (A1 and E4, Fig. 2d) in regions A and E, suggesting that the ITH was more extensive on the single-cell level, and the depth (~100×) of the WES used in the multi-region WES was insufficient to capture all of the low-frequency mutations present in minor subclones.

### Single-cell sequencing revealed de novo focal SCNAs that were hidden in the bulk sequencing

**Fig. 3:**
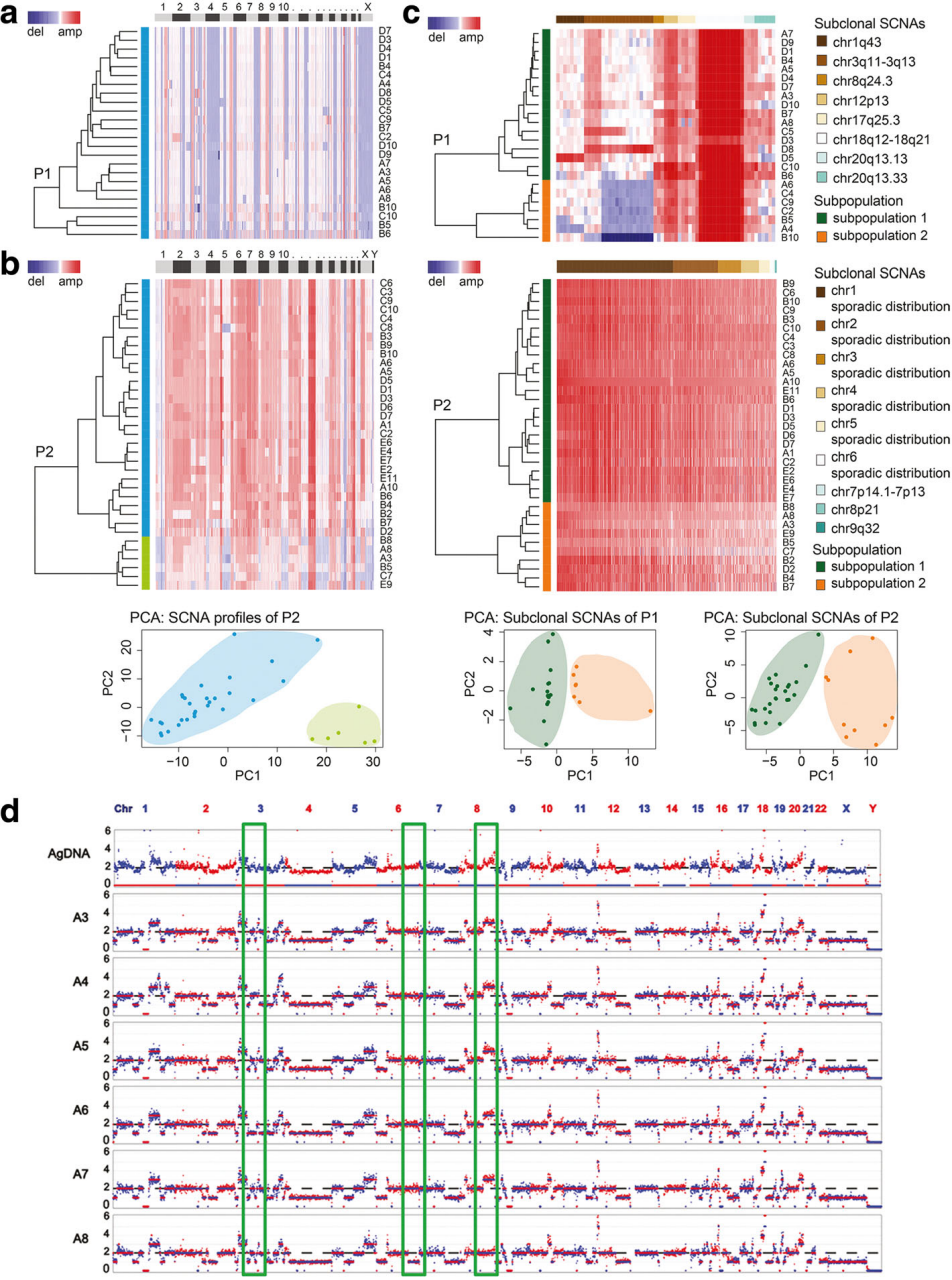
Single-cell sequencing showed more subtle differences than multi-region WES. **a** Clustered heatmap of 24 single tumor cells with SCNA profiles in patient 1 based on Euclidean distance and ward.D method. The x axis was plotted by chromosomes from chr1 to chrX/Y and the y axis was the population labeled by blue. **b** Clustered heat map and PCA of 35 single tumor cells of patient 2 based on SCNA profiles. Single tumor cells were grouped into two clusters. The x axis was plotted by chromosomes from chr1 to chrX/Y and the y axis was subpopulations labeled by blue and green. **c** Subclonal SCNAs of patients 1 and 2 divided single tumor cells into two subpopulations, which was in accordance with two clusters identified by PCA. The chromosomes (columns) where subclonal SCNAs more than 1.5 Mb located was showed in colors. The two subpopulations (rows) were labeled in colors. **d** Single tumor cells showed more differences in regional level than gDNA in reigon A of patient 1. P1: patient1; P2: patient 2

After excluding all the diploid cells and one cell doublet (single cell D2 of patient 1) from further analyses, clustering analyses based on large-scale SCNA profiles showed that there was one population in patient 1 (Fig. 3a), whereas two subpopulations were detected in patient 2 (Fig. 3b). PAM clustering [39] also supported two subpopulations of patient 2 (Additional file 1: Fig. S4).

We further analyzed the single cell SCNA data with PCA. The subclonal SCNAs of single cells were identified by PCA based on the depth of each bin. The subclonal SCNAs of more than 1.5 Mb in patient 1 (63 bins) were visualized with a clustered heatmap (Fig. 3c, Additional file 1: Fig. S5). In stark contrast to the large-scale copy number-based clustering, single cells of patient 1 were clustered into two groups based on subclonal SCNAs (>1.5 Mb), supported by PAM clustering [39] which also quantified two clusters (Additional file 1: Fig. S4). The subclonal SCNAs of patient 2 were more extensive and complicated (1674/6037 bins before manual selection), which might be related with the advanced stage. Based on the large-scale copy number-based clustering, the PCA of patient 2 confirmed the existence of two subpopulations (Fig. 3b). The single tumor cells of patient 2 were also clustered into two groups based on subclonal SCNAs (806 bins), though the proportion of two subpopulation altered from 29:6 to 25:10, meaning that the preponderant subpopulation based on the large-scale copy number-based clustering might divided into two subclones because of subclonal SCNAs (29 = 25 + 4) in the future (Fig. 3c, Additional file 1: Fig. S5). The PAM results [39] also supported two clusters existed (Additional file 1: Fig. S4). These results implied that single tumor cells had different fitness advantages owing to subclonal SCNAs, and could possibly form more subpopulations at a later stage during tumor progression.

The SCNA profiles of genomic DNA extracted from multiple regions were distorted by the presence of somatic diploid cells, whereas the profiles obtained by the sequencing of single tumor cells likely revealed the true differences within the bulk tumor. Therefore, single-cell sequencing is necessary to precisely determine the true number of different subclones within a tumor cell population [40]. For instance, variable SCNAs in certain chromosomal regions in single tumor cells were hidden in the bulk gDNA in region A of patient 1 (Fig. 3d). The frequencies of the two subpopulations based on SCNA profiles in patient 2 were 17% (6/35) and 83% (29/35). The SCNA-based subclonal frequencies of patient 2 might explain the regional differences observed in the multi-region WES (Additional file 1: Fig. S3), which arose from the proportions of the two subpopulations in each region.

### Differences between the two patients

**Fig. 4:**
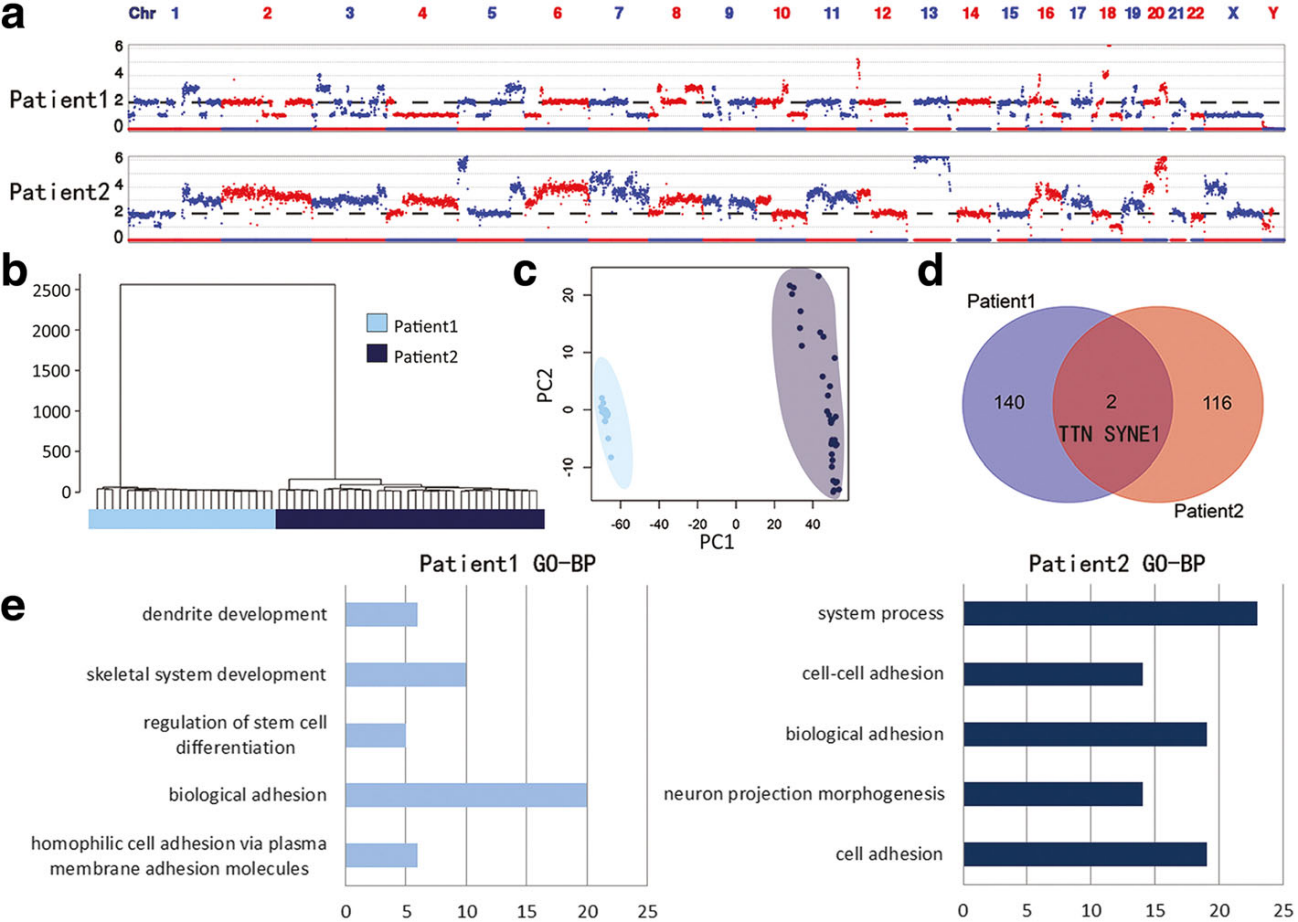
Individual differences between two patients. **a** The consensus copy number profiles of two patients. Each patient had a specific individual large-scale copy number pattern. **b** The hierarchical clustering using Euclidean distance and ward.D method showed that single tumor cells were grouped into two populations according to two patients. **c** The PCA showed that single tumor cells were divided into two clusters according to two patients. **d** The Venn diagram of mutations from two patients. Two patients merely had TTN and SYNE1 mutated genes in common. **e** GO-BP analyses of mutated genes in two patients. The top five biological processes of the two patients were quite different and x axis was labeled by the number of mutated genes involved in each process, *p* < 0.05

We evaluated the ITH of two rectal cancer patients at the multi-region and single-cell levels. Each patient showed unique large-scale copy number patterns (Fig. 4a). Hierarchical clustering and PCA showed that 24 tumor cells of patient 1 and 35 tumor cells of patient 2 were obviously grouped into two populations (Fig. 4b and c). The two patients only had TTN and SYNE1 mutations in common (Fig. 4d), and these genes might play a role in chromosome segregation during mitosis [41] and subcellular spatial organization [42]. Gene Ontology (GO) terms based on biological processes (DAVID 6.7) showed that the mutated genes in patient 1 were clustered in homophilic cell adhesion via plasma membrane adhesion molecules, biological adhesion, and regulation of stem cell differentiation, while the mutated genes in patient 2 were clustered in cell adhesion, neuron projection morphogenesis, and biological adhesion (Fig. 4e). In a word, the copy number profiles and mutational scenarios of the two patients were quite different, suggesting the necessity of personalized medicine in clinical therapy.

## Discussion

In this study, we performed multi-region integrated single-cell sequencing to explore the ITH in two rectal tumors. The large-scale copy number profiles of multiple regions and single tumor cells in each patient appeared to be similar, implying that the majority of chromosomal rearrangements were early events and were inherited clonally and steadily, which was accordant with previous studies on breast cancer [12, 13]. Besides the clonal SCNAs, some subclonal SCNAs were also observed by single-cell sequencing. Subclonal SCNAs, which are generated by later events during tumorigenesis, play an important role in boosting single-cell heterogeneity. In the mutational scenarios, the ubiquitous mutations are formed early in tumor-initiating cells and are inherited by their offspring, whereas the “private” mutations accumulate sporadically and markedly increase the ITH among different individuals. Subclonal SCNAs and sporadic mutations might impart further advantages to certain subpopulations during tumor growth and mutually facilitate the ITH.

We applied 40 single cells and 48 single cells to evaluate the ITH for patients 1 and 2, respectively. After removing the diploid somatic cells, there were 24 and 35 tumor cells with SCNAs for patients 1 and 2, respectively. A previous study on breast cancer suggested that 20-40 single cells were eligible for detecting SCNA-based subpopulations [13], which was compatible with our results about subclonal SCNAs. Therefore, the amount of single cells for each patient we have studied was reasonable. The computationally derived tumor percentage of each region was determined by Sequenza (Additional file 1: Fig. S3). The separated regions of one tumor were assessed by the pathologists, of which the histological features were reckoned similar. The tumor purifies of two patients identified by the pathologists were both more than 90%, but the deduced results of WES showed that the tumor purity of P1 was just 25-49% (Additional file 1: Fig. S3) owing to somatic cell infiltration. The lower tumor purity of P1 might give rise to lower ITH in some extent, since the diploid cell contamination would mask the true profiles, distorting the SCNA profiles and descending the mutational heterogeneity by missing low frequency mutations. When obtaining the tumor mutations by WES, the germline mutations could be excluded by comparing tumor regions to peripheral blood or normal rectum samples. Here, we utilized peripheral blood but not normal rectum as control in order to avoid missing somatic mutations that existed early in both adjacent normal tissues and tumors, which is rare but could happen in some cases.

The heterogeneity of distinct regions of one tumor arises from the proportion of various subclones. Tumor tissue is a mixture of different cell populations that interact with the microenvironment, and the evolution of tumorigenicity is complex and dynamic. The preponderant subclone adapting to the circumjacent microenvironment plays a dominant role in certain region of one tumor, of which the master status is dynamically changing. For instance, though substantial tumor cells could be killed during the therapy, there were still survival of rare subclones with resistance to drugs, which might lead to relapse. It is the heterogeneity that make some tumors so hard to eradicate. At single-cell level, SCNAs were confirmed to be in correlation with gene expression [43], and the SCNAs of colorectal cancer, which affected the expression of functional genes, were reported to be potential biomarkers [35]. For instance, there was only one population according to the large-scale copy number profiles in patient 1, but when zoom in to focal SCNA alterations, there were apparently two subpopulations, meaning that although the large-scale copy number profiles (24 chrmosomes) appear to be similar at this time snap-shot, the single tumor cells possibly form two subpopulations owing to the differences in subclonal SCNAs in the future. Besides clonal SCNAs which all tumor cells steadily inherited, subclonal SCNAs would facilitate further cell-to-cell heterogeneity, which might lead to different therapy requirement. Among the subclonal SCNAs in patient 1, *MINA*, which is located in the focal region chr3q11.2, is a c-Myc target gene that may affect cell proliferation [44]. The tumor suppressor genes *PIK3C3* on chr18q12.3 and *SMAD2* on chr18q21.1, which affected the TGF-β pathway, were reported to be related to metastasis [35, 45]. SCNAs induced upregulation or downregulation of these important genes would eventually give rise to growth advantages in certain populations during tumor progression.

Two patients were of the same age, no smoking, no alcohol intake, and both adenocarcinoma without microsatellite instable. The protein biomarkers of two tumors were different, CEA was highly expressed in P1, while CA72.4 was highly expressed in P2. Even though P2 (T3), which had one lymph node metastasis and positive nerve invasion, was further progressed than P1 (T2), the postoperative therapy was quite effective. The regular follow-up showed that the two patients under personalized medicine were healthy with no relapse after surgery. Consistent with previous studies [46], our study also demonstrated the mutational diversification of multiple regions and branch evolution in rectal cancer. Additionally, we found that the regional differences in SCNA profiles of different tumor regions might arise from different subpopulations (Fig. 3a and b). Single-cell sequencing further confirmed the distributions of minor subpopulations, and revealed the subclonal structure of the tumor. Minor cell populations might exist early in tumorigenesis but in limited quantities, or they might be generated later with extraordinary growth advantages [47].

Tumors are composed of many cells, and bulk sequencing only reveals the average genomic alterations of this cell mixture; thus, clonal analysis cannot resolve the subclonal composition of a tumor beyond the resolution of the sample used for the analysis. Contamination by diploid cells and the proportions of tumor subpopulations may affect the SCNA profiles of tumor regions. Moreover, deep sequencing is required to detect rare mutations in bulk tumor, which is costly. Thus, single-cell sequencing is of significant importance in investigating tumor cell heterogeneity and in discovering subtle diversification. However, it should be noted that we did not find any correlation between the copy number variation and mutation events. In accordance with the previous report [48], our results also suggest that a single biopsy is sufficient for determination of major copy number profiles and high-frequency mutations for target therapy, however, it is insufficient for precise detection of subclonal SCNAs and low-frequency mutations.

In a conclusion, although the two patients are of the same molecular classification, the extent of heterogeneity differed. There are different clinicopathological features and molecular paths of tumorigenesis in colon and rectal cancer [3], so it is meaningful to focus just on rectal tumors. Personalized medicine, tailored to each individual based on druggable genes, is necessary. In addition, the extensive ITH might also indicate that there are many possibilities for drug resistance in each patient. This study provides a preliminary impression of ITH in rectal cancer.

## Conclusions

The SCNA profiles of multiple regions and single tumor cells within one tumor are similar, suggesting that a considerable number of SCNAs are early events in cancer development and inherited steadily. The regional differences of SCNA profiles within multiple regions arise from different proportions of SCNA-based subpopulations. Single-cell WGS shows focal SCNAs that were not detected in the multi-region WES, implying that a detailed genetic characterization of the tumor can be better uncovered by single-cell sequencing. Although the two patients are of the same molecular classification, the extent of heterogeneity differed. Intertumor heterogeneity supports the necessary of personalized medicine tailored to each patient based on clonal target genes. Intratumor heterogeneity means there are many possibilities for drug resistance in each patient.

## Additional files

Additional file 1: **Figs. S1-S5** and **Tables S1-S6**. (DOCX 1794 kb)

## Abbreviations

COSMIC: Catalog of Somatic Mutations in Cancer
CTCs: Circulating tumor cells
FACS: Fluorescent activated cell sorting
ITH: Intratumor heterogeneity
MAPD: Median of the absolute values of all pairwise differences
PAM: Partition around medoids
PCA: Principle component analysis
SCNAs: Somatic copy number alterations
SNVs: Single nucleotide variations
VAF: Variant allelic frequency
WES: Whole-exome sequencing
WGS: Whole-genome sequencing
