# Supplementary material for: Multi-region and single-cell sequencing reveal variable genomic heterogeneity in rectal cancer
Source: BMC Cancer. 2017 Nov 23;17:787. doi: 10.1186/s12885-017-3777-4 (PMC5701298; doi:10.1186/s12885-017-3777-4)
Supplement: Supplementary file 1 — Figs. S1-S5 and Tables S1-S6. (DOCX 1794 kb) [file 12885_2017_3777_MOESM1_ESM.docx]

## Figure S1


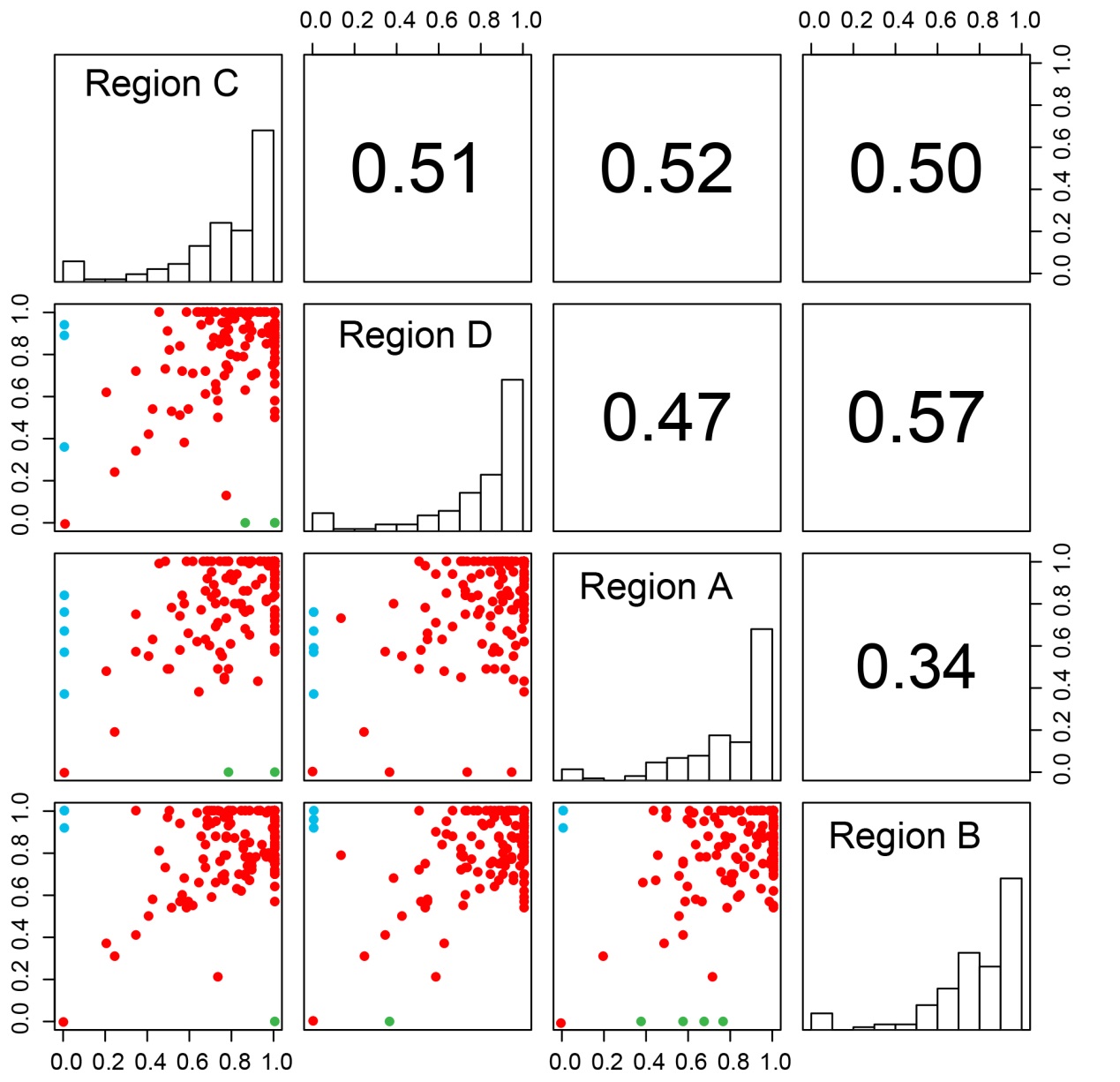


**Figure S1. Multiple plots showed mutational diversity of multi-region WES in patient 1.** On the [scatter](http://www.baidu.com/link?url=CSwq2taI07C55c5z5DvC3lsfXtXC1GUBhk1Fv5dKaSYSBULJdqpzY5gFZwrbomEY2lmZFYItPJJD23-mmEZiH-VrzTZoWi_6lCXwHD4laJ41XM8n6nrxaylSysbxhL50) plots of the cancer cell fraction (CCF) in the left, red dots represented mutations shared by two regions, while blue and green dots on axes represented mutations which were region-specific mutations. The *x* and *y* axes showed the CCF values. The histograms in the middle showed the density of mutations with different CCF values in each region. On the right, each number in the box represented the correlation coefficient (pearson correlation) between two samples.

## Figure S2


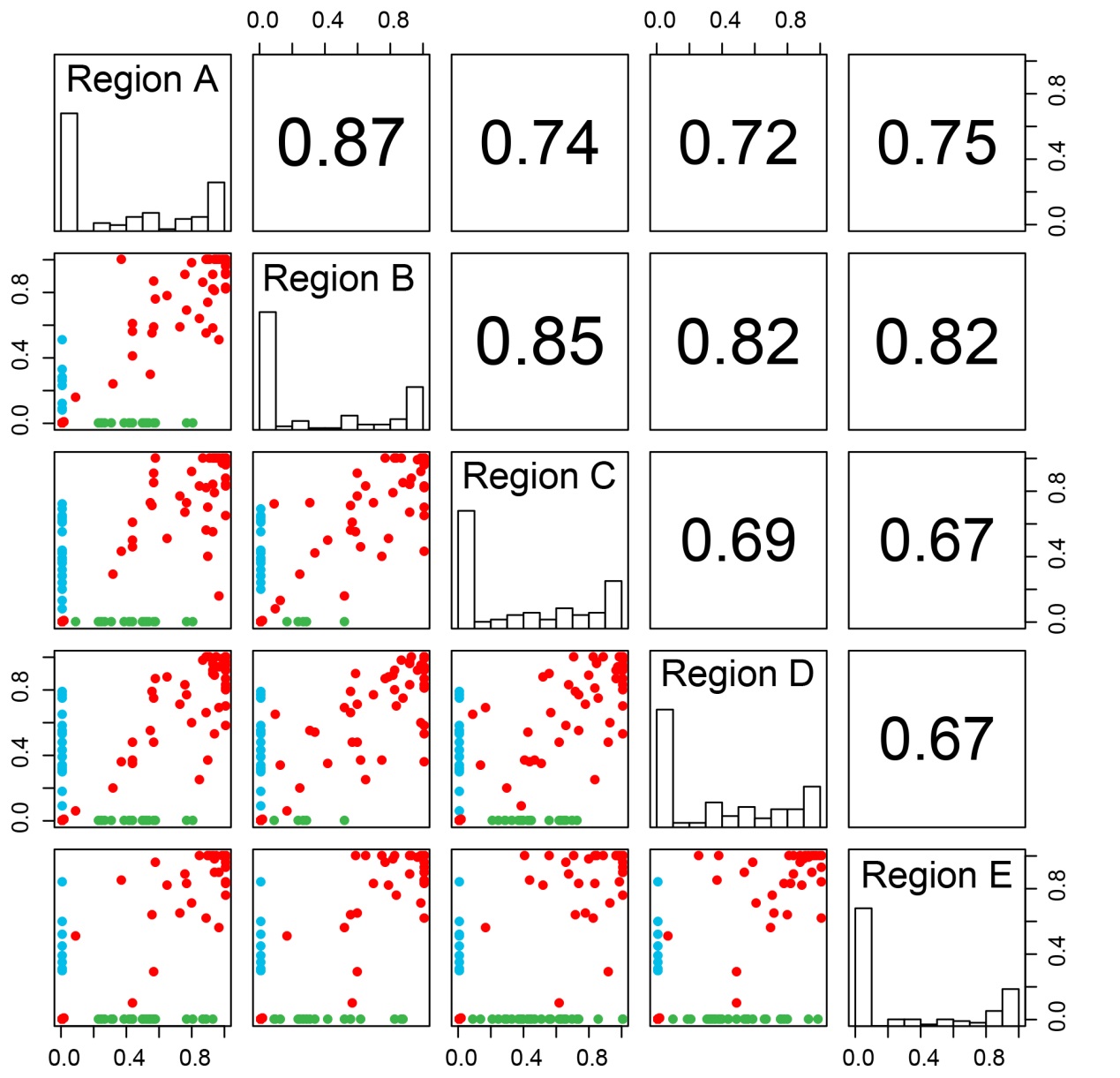


**Figure S2. Multiple plots showed mutational diversity of multi-region WES in patient 2.** On the [scatter](http://www.baidu.com/link?url=CSwq2taI07C55c5z5DvC3lsfXtXC1GUBhk1Fv5dKaSYSBULJdqpzY5gFZwrbomEY2lmZFYItPJJD23-mmEZiH-VrzTZoWi_6lCXwHD4laJ41XM8n6nrxaylSysbxhL50) plots of the cancer cell fraction (CCF) in the left, red dots represented mutations shared by two regions, while blue and green dots on axes represented mutations which were region-specific mutations. The *x* and *y* axes showed the CCF values. The histograms in the middle showed the density of mutations with different CCF values in each region. On the right, each number in the box represented the correlation coefficient (pearson correlation) between two samples. **T**he multiple regions of patient 2 were more heterogeneous than those of patient 1, since the blue and green dots were much more in patient 2, which were referred to region-specific mutations. The mutations with CCF=0 is too much more in patient 2, so the correlation coefficients of patient 2 is higher than those of patient 1.

## Figure S3

Patient 1 SCNA profiles inferred with WES data


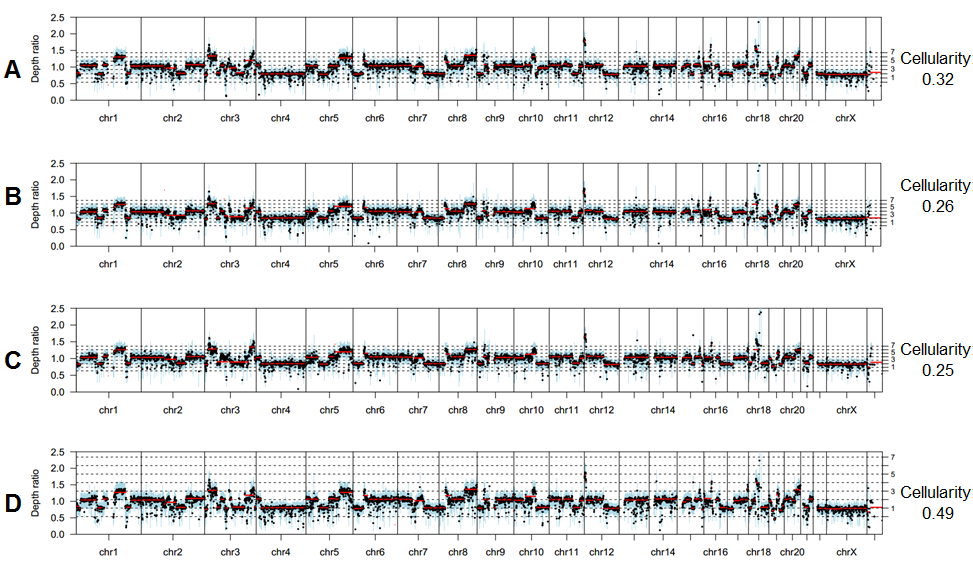


Patient 2 SCNA profiles inferred with WES data


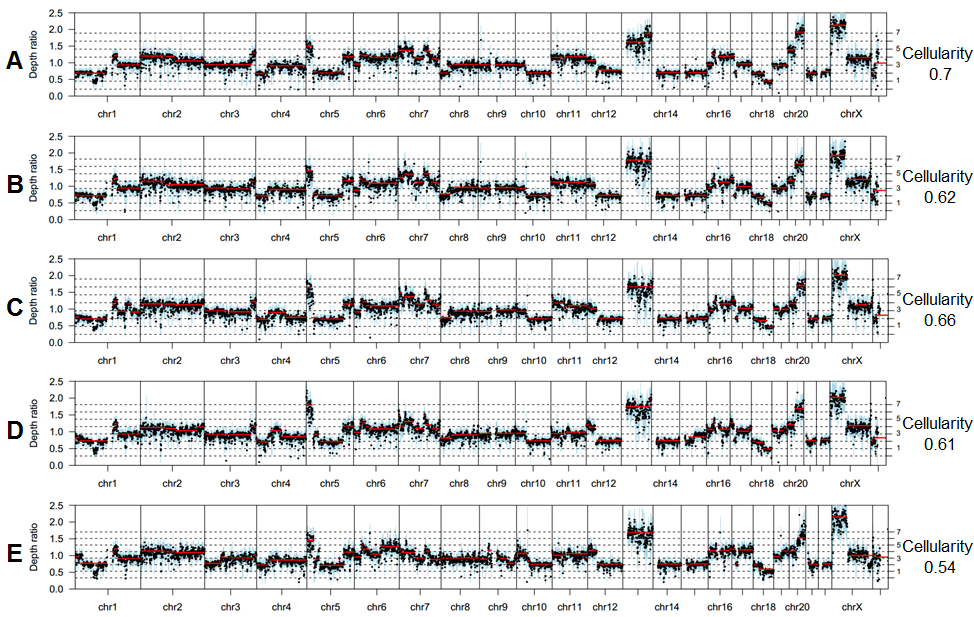


**Figure S3. The SCNA profiles and tumor cellularity of multi-region samples inferred by Sequenza.** The SCNA profiles inferred by Sequenza based on whole-exome sequencing showed that there were obvious differences among the five regions in patient 2. Cellularity represented the purity of tumor cells within each region.

## Figure S4


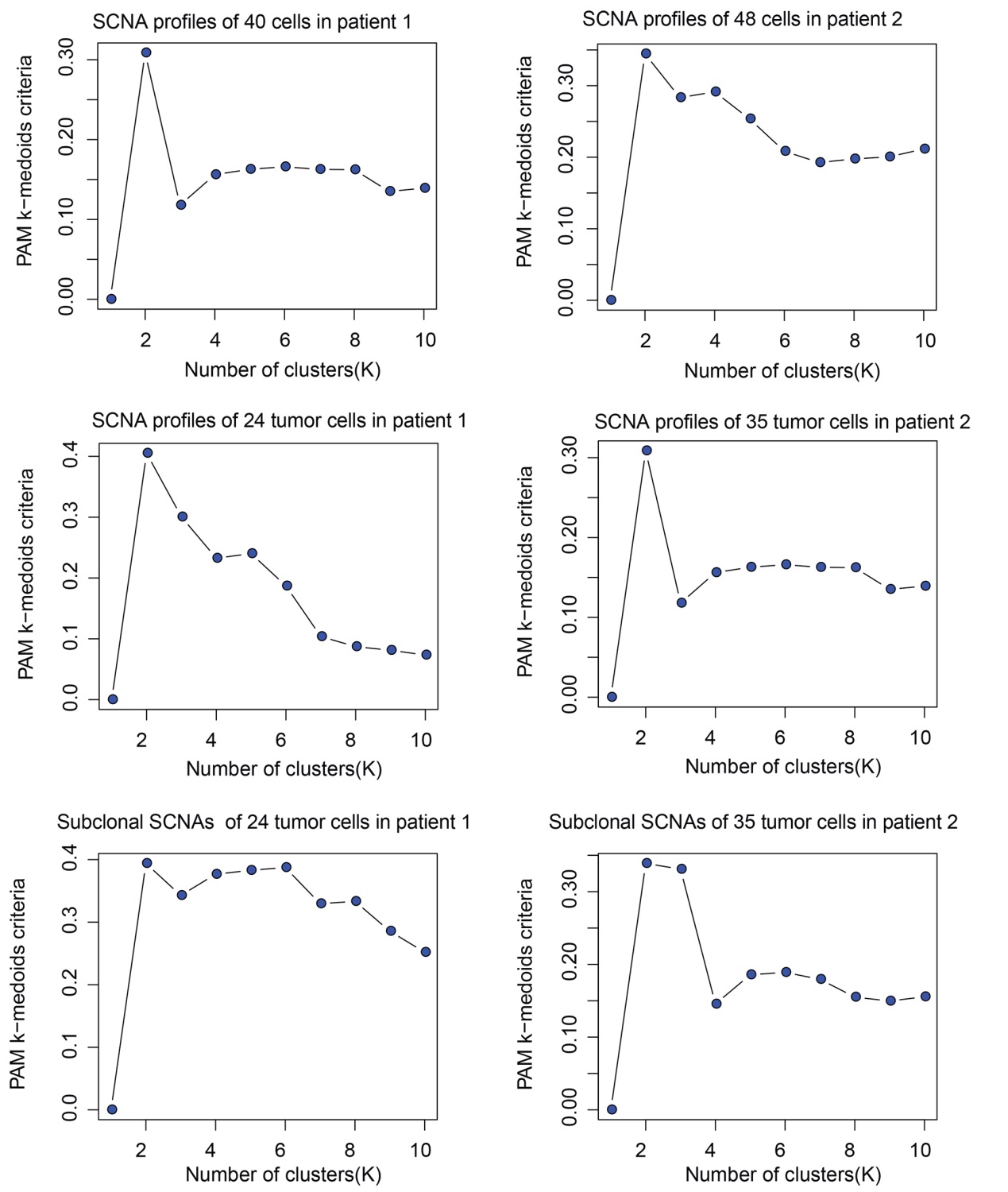


**Figure S4. Partitioning around medoids (PAM) clustering quantified the number of clusters in two patients.** PAM clustering was performed with the fpc package with criterion “asw” of the pamk function. The number of x axis where highest peak located indicated the optimal number of clusters. The PAM results supported most of the hierarchical clusterings except for 24 tumor cells of patient 1 based on large-scale SCNA profiles, which we preferred to be one population.

## Figure S5


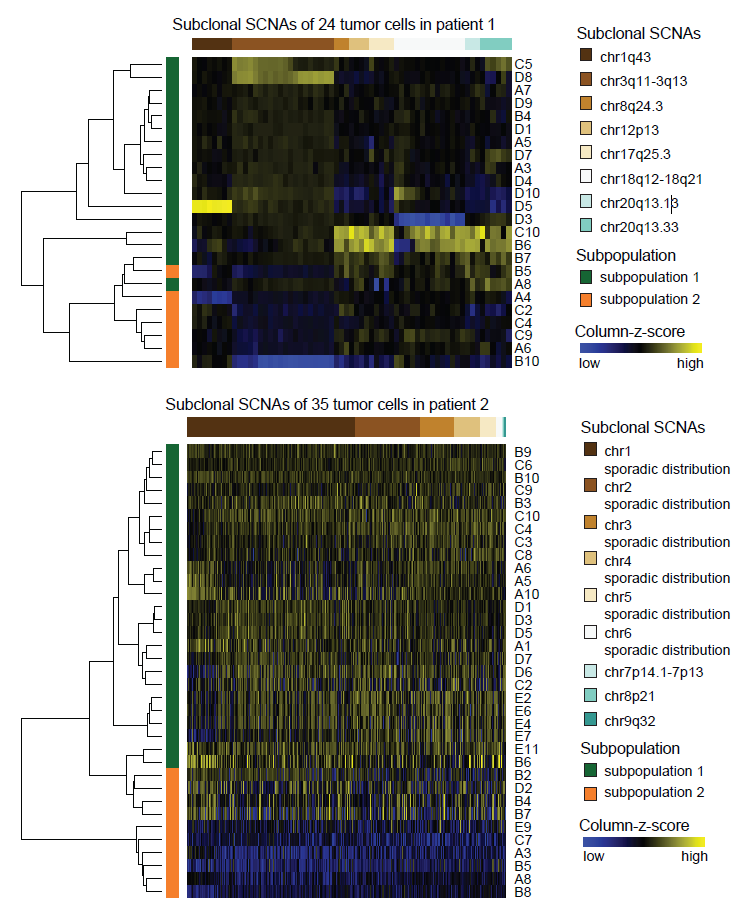


**Figure S5. The heterogeneity of subclonal SCNAs in patients 1 and 2.** The chromosomes (columns) where subclonal SCNAs more than 1.5Mb located was showed in colors. The two subpopulations (rows) were labeled in colors. We set column-z-score to exhibit the discrepancy of each bin in single tumor cells, the relatively gains labeled in yellow and the losses in blue for each bin. The hierarchical clustering was based on Euclidean distance and ward.D method.

**Table S1. Clinicopathological characteristics of the two patients**

|  | **Patient 1** | **Patient 2** |
| --- | --- | --- |
| Age | 50-60 | 50-60 |
| Gender | Female | Male |
| Tumor location | Rectum | Rectum |
| Depth of invasion | T2 | T3 |
| Lymph node metastasis | N0 | N1 (1/17) |
| TNM Stage | I | IIIB |
| Tumor size(cm) | 3.0x2.0x1.2 | 5.5x4.5x0.6 |
| Macroscopic type | Fungoid | Ulcerative |
| Histology | Adenocarcinoma | Adenocarcinoma |
| Differentiation | Moderate-Poor | Moderate |
| Nerve invasion | Negative | Positive |
| Vascular thrombus | Negative | Positive |
| Microsatellite instable | Negative | Negative |
| Smoking | None | None |
| Alcohol intake | None | None |
| Previous removal of benign tumors | No | No |
| Relapse after surgery | No | No |
| CEA (0-5 ng/ml) | 17.03 ↑ | 4.07 |
| CA199 (0-37 U/ml) | 10.78 | 16.12 |
| CA72.4 (0-6.7 U/ml) | 1.00 | 11.22 ↑ |

**Table S2. Primers for Sanger Sequencing.**

| Patient | Gene | Forward primer | Reverse primer |
| --- | --- | --- | --- |
| P1 | ATM | TGGATGGCATCTGCTCTATTT | CAAGAAGTGGCAGAGTCAGTAT |
|  | ATM-2 | GAGGTGTTCTTGTGACAAACAG | AGGCTTAAGCTTCAGACCATAA |
|  | C9 | GGCAGTGCTCATTGACATCTA | AGGAACCTAAGTAAGGATGTTGC |
|  | CUL9 | GAGAGCCATACCCTCACCT | AGAGGCTGAGGAAGGACAA |
|  | EP300 | GGTATCTATATCAACTCCAACTTGTG | CTATGTCCCAACTGACCTTGTA |
|  | GPR139 | AGCAGTAGAGGAAGAAGTTGATG | GTGTACAAGCTCAGGAGGAAG |
|  | GPR98-1 | GAGAGCAACTCAAGGAAGAGAC | GTCTGGCTTCCAGTTACCTTAC |
|  | GPR98-2 | GATTGTAACCCTCACCCGTATC | GAGTATGACCTGCCAAACTGAA |
|  | MUC16-1 | TCTTGTGACATTGTGGACTGAT | CATACCTGCTTCCTCTGAGATAAC |
|  | MUC16-2 | AGTCATAGGAGAGGAGAGCTTAT | GGGCTACATCACAGGATACTT |
|  | PAPPA2 | GTGACTCCTTAGAGGCTGAAAT | CGTGTAGCTCATGTAGTTGGT |
|  | PCDH15 | GTCCAACAAATTGAGGAAGCAA | GTAATGCGGCTGAATGAAACTC |
|  | SOX5 | TGGGCTGTTTGTGCTCTT | ATGAAATCTGAGGTGACAGCTT |
|  | TG | TTGGAGCCTGGCACATAATAG | GCCAAGAGTAGAAGGAAGGAAG |
|  | TMPRSS11A | GAGATACCCACCACCATAGTAAAG | GATAAGGCTCAGCACACAGAT |
| P2 | ALOX5 | TGAATCACTGGCAGGAAGAC | GACTCGAGCATCATGGAGAAA |
|  | APC | GGGTTCAACTACACGAATGGA | GCCACCACACTGGCTAAA |
|  | C6 | GGCTTGTTTGAAGGCAGAATTA | CCCTCAGTGCAGGAAATAACA |
|  | ERBB2 | TGAGCCCAGGCAGAAATAAG | CAGGCAGAGGGTGAGATTAAAG |
|  | NOD2 | AGGCATCTGCAAGCTCATT | GGGAGATCACAGCATTAGAGAAC |
|  | NUP98 | GCTATGATCCCACCATTGTACTC | ATGGAGTTTGGCCAACAGATAA |
|  | PAPPA | CAGTTGGAAAGCGATGAGTCTA | GGGTCAGTGGGCTGAATATG |
|  | PDE11A | GTGCATCCTTGAGATGTTAGGT | GAAGCCGATGCTAGATTCAGTAG |
|  | PRKCG | GAGAACCTGAGAAGGAGAGAGA | TCACAGAGAAGGATGAGACAGA |
|  | RNF213 | TTTGCTAGAGACAGGGTTTCG | TCTCTCCAAAGTCATCCAAAGATAG |
|  | SAMD9L | CTCTGCCCAGCAGTGTATTAAA | CCCGTGGATCTTCTTCAGTTATC |
|  | SHC1 | CCTATCTCGGCTCCCTTCATA | CCTGCCCACCACACATTTA |
|  | TP53 | AGGTGGATGGGTAGTAGTATGG | CATCTTGGGCCTGTGTTATCT |
|  | TTN | CCGTCATAGGTGGGTTTCTTC | CAACCACCTGGCAAATTGTATC |
|  | VDR | GGACACTCACACTCCTTCATC | GCCTCATGTCTTCTGTTGGA |

# Table S3. Sanger sequencing validated diploid cells in each patient.

| Patient 1 | ATM  G-T | TMPRSS11A  G-A | PCDHB15  G-A | TG  C-T | PAPPA2  C-A |
| --- | --- | --- | --- | --- | --- |
| A1 | G | G | - | C | C |
| A2 | G | G | - | - | C |
| A9 | G | G | - | - | C |
| A10 | G | G | - | C | C |
| B1 | T | A | - | T | A |
| B2 | G | G | - | C | - |
| B3 | G | G | - | C | - |
| B8 | G | G | - | C | C |
| B9 | G | G | - | - | - |
| C1 | - | G | - | C | C |
| C3 | G | G | - | C | - |
| C6 | G | G | - | C | C |
| C7 | G | G | G | - | C |
| C8 | - | A | G | - | - |
| D6 | G | G | G | - | C |
| Bulk tumor | T | A | A | T | A |

| Patient 2 | APC  C-T | ERBB2  C-T | TP53  C-T | ALOX5  G-A | TTN  C-A | PAPPA  G-A |
| --- | --- | --- | --- | --- | --- | --- |
| A2 | C | C | C | G | C | G |
| A4 | C | C | C | G | C | G |
| A7 | C | C | - | - | - | G |
| A9 | C | C | C | - | C | - |
| B1 | C | C | C | - | C | - |
| C1 | C | C | - | - | C | G |
| C5 | C | C | - | - | - | - |
| D4 | C | C | C | - | - | G |
| E1 | C | C | - | - | - | G |
| E3 | C | C | - | - | C | G |
| E5 | C | C | C | - | - | G |
| E8 | C | C | C | - | C | G |
| E10 | C | C | - | - | C | G |
| Bulk tumor | T | T | T | A | A | A |

*Gene wildtype to mutant *Bulk tumor: the genomic DNA of bulk contained wildtype and mutant of each gene simultaneously.

**Table S4. Summary of whole-exome sequencing information**

| ID | Samples | Average Depth | Target Covered > 10× | Target Covered > 20× |
| --- | --- | --- | --- | --- |
| Patient 1 | Region A | 112.37× | 98.6% | 95.9% |
|  | Region B | 116.91× | 98.7% | 96.4% |
|  | Region C | 110.82× | 98.6% | 96% |
|  | Region D | 111.36× | 98.9% | 96.7% |
|  | Blood | 98.33× | 98.5% | 95.5% |
| Patient 2 | Region A | 122.58× | 98.6% | 96% |
|  | Region B | 105.25× | 98.5% | 95% |
|  | Region C | 116.84× | 98.5% | 95.3% |
|  | Region D | 117.38× | 98.5% | 95.4% |
|  | Region E | 120.52× | 98.9% | 96.2% |
|  | Blood | 104.61× | 98.5% | 95.7% |

# Table S5. Summary of synonymous and nonsynonymous somatic mutations identified in two patients.

**Patient 1**

| Chr | Position | Wildtype | Mutant | Change type | Gene name | Effect | AA Old/New | Condon Old/New |
| --- | --- | --- | --- | --- | --- | --- | --- | --- |
| 1 | 17281988 | G | A | SNP | CROCC | NON_SYNONYMOUS_CODING | R/Q | cGg/cAg |
| 1 | 29602269 | G | A | SNP | PTPRU | SPLICE_SITE_DONOR | - | - |
| 1 | 35453709 | C | A | SNP | ZMYM6 | NON_SYNONYMOUS_CODING | G/C | Ggt/Tgt |
| 1 | 35885138 | A | C | SNP | ZMYM4 | NON_SYNONYMOUS_CODING | S/R | Agc/Cgc |
| 1 | 39776007 | G | T | SNP | MACF1 | NON_SYNONYMOUS_CODING | V/L | Gtg/Ttg |
| 1 | 49242286 | G | A | SNP | BEND5 | NON_SYNONYMOUS_CODING | A/V | gCg/gTg |
| 1 | 55151997 | C | G | SNP | HEATR8 | NON_SYNONYMOUS_CODING | P/A | Cct/Gct |
| 1 | 114391229 | T | C | SNP | PTPN22 | NON_SYNONYMOUS_CODING | Y/C | tAc/tGc |
| 1 | 149882153 | G | A | SNP | SV2A | NON_SYNONYMOUS_CODING | T/M | aCg/aTg |
| 1 | 153662438 | C | T | SNP | NPR1 | NON_SYNONYMOUS_CODING | R/C | Cgc/Tgc |
| 1 | 156894222 | C | T | SNP | LRRC71 | NON_SYNONYMOUS_CODING | P/L | cCg/cTg |
| 1 | 176659409 | C | A | SNP | PAPPA2 | STOP_GAINED | C/* | tgC/tgA |
| 1 | 205899103 | C | T | SNP | SLC26A9 | NON_SYNONYMOUS_CODING | G/S | Ggc/Agc |
| 1 | 220338127 | A | C | SNP | RAB3GAP2 | NON_SYNONYMOUS_CODING | F/L | ttT/ttG |
| 2 | 5832939 | G | T | SNP | SOX11 | NON_SYNONYMOUS_CODING | C/F | tGc/tTc |
| 2 | 27150238 | G | A | SNP | DPYSL5 | NON_SYNONYMOUS_CODING | A/T | Gct/Act |
| 2 | 68609686 | G | T | SNP | PLEK | NON_SYNONYMOUS_CODING | L/F | ttG/ttT |
| 2 | 119600825 | G | A | SNP | EN1 | NON_SYNONYMOUS_CODING | R/C | Cgc/Tgc |
| 2 | 130912761 | C | T | SNP | SMPD4 | NON_SYNONYMOUS_CODING | R/H | cGc/cAc |
| 2 | 179648990 | G | T | SNP | TTN | NON_SYNONYMOUS_CODING | A/D | gCt/gAt |
| 2 | 202574667 | C | A | SNP | ALS2 | NON_SYNONYMOUS_CODING | R/M | aGg/aTg |
| 2 | 220290456 | C | T | SNP | DES | NON_SYNONYMOUS_CODING | R/W | Cgg/Tgg |
| 2 | 223786061 | C | A | SNP | ACSL3 | NON_SYNONYMOUS_CODING | S/Y | tCc/tAc |
| 2 | 238249551 | C | T | SNP | COL6A3 | NON_SYNONYMOUS_CODING | A/T | Gcg/Acg |
| 3 | 14555937 | C | T | SNP | GRIP2 | NON_SYNONYMOUS_CODING | R/Q | cGg/cAg |
| 3 | 32859909 | G | A | SNP | TRIM71 | NON_SYNONYMOUS_CODING | A/T | Gcc/Acc |
| 3 | 38040481 | A | T | SNP | VILL | STOP_GAINED | K/* | Aag/Tag |
| 3 | 38991804 | C | A | SNP | SCN11A | NON_SYNONYMOUS_CODING | R/L | cGc/cTc |
| 3 | 48694146 | G | A | SNP | CELSR3 | NON_SYNONYMOUS_CODING | R/C | Cgc/Tgc |
| 3 | 53289892 | G | A | SNP | TKT | NON_SYNONYMOUS_CODING | R/C | Cgt/Tgt |
| 3 | 57457329 | A | G | SNP | DNAH12 | NON_SYNONYMOUS_CODING | I/T | aTt/aCt |
| 3 | 85932519 | C | A | SNP | CADM2 | NON_SYNONYMOUS_CODING | S/Y | tCt/tAt |
| 3 | 122350994 | G | C | SNP | PARP15 | NON_SYNONYMOUS_CODING | E/D | gaG/gaC |
| 3 | 172834965 | C | A | SNP | SPATA16 | NON_SYNONYMOUS_CODING | C/F | tGc/tTc |
| 3 | 194080610 | A | G | SNP | LRRC15 | NON_SYNONYMOUS_CODING | V/A | gTc/gCc |
| 4 | 68784778 | G | A | SNP | TMPRSS11A | NON_SYNONYMOUS_CODING | R/C | Cgc/Tgc |
| 4 | 89052266 | G | A | SNP | ABCG2 | NON_SYNONYMOUS_CODING | R/W | Cgg/Tgg |
| 4 | 22750457 | * | -TAG | DEL | GBA3 | SPLICE_SITE_ACCEPTOR | - | - |
| 5 | 5242231 | G | T | SNP | ADAMTS16 | NON_SYNONYMOUS_CODING | E/D | gaG/gaT |
| 5 | 21752076 | G | T | SNP | CDH12 | NON_SYNONYMOUS_CODING | Q/K | Caa/Aaa |
| 5 | 39308403 | G | T | SNP | C9 | NON_SYNONYMOUS_CODING | A/D | gCt/gAt |
| 5 | 89979992 | C | T | SNP | GPR98-1 | NON_SYNONYMOUS_CODING | A/V | gCg/gTg |
| 5 | 90049635 | C | A | SNP | GPR98-2 | NON_SYNONYMOUS_CODING | A/E | gCa/gAa |
| 5 | 137761136 | T | A | SNP | KDM3B | NON_SYNONYMOUS_CODING | S/T | Tct/Act |
| 5 | 140035507 | A | G | SNP | IK | NON_SYNONYMOUS_CODING | D/G | gAt/gGt |
| 5 | 140530972 | G | T | SNP | PCDHB6 | NON_SYNONYMOUS_CODING | Q/H | caG/caT |
| 5 | 140625822 | G | A | SNP | PCDHB15 | NON_SYNONYMOUS_CODING | V/I | Gtc/Atc |
| 5 | 161300330 | C | A | SNP | GABRA1 | NON_SYNONYMOUS_CODING | L/M | Ctg/Atg |
| 6 | 43188564 | G | A | SNP | CUL9 | NON_SYNONYMOUS_CODING | R/H | cGc/cAc |
| 6 | 55625314 | G | T | SNP | BMP5 | NON_SYNONYMOUS_CODING | Q/K | Caa/Aaa |
| 6 | 97423901 | C | A | SNP | KLHL32 | NON_SYNONYMOUS_CODING | L/I | Ctc/Atc |
| 6 | 108279142 | * | +C | INS | SEC63 | FRAME_SHIFT | - | - |
| 6 | 109768314 | G | A | SNP | MICAL1 | NON_SYNONYMOUS_CODING | T/I | aCa/aTa |
| 6 | 123658808 | C | A | SNP | TRDN | NON_SYNONYMOUS_CODING | R/I | aGa/aTa |
| 6 | 147103265 | A | T | SNP | ADGB | NON_SYNONYMOUS_CODING | K/N | aaA/aaT |
| 6 | 151917469 | G | T | SNP | CCDC170 | SPLICE_SITE_ACCEPTOR | - | - |
| 6 | 152737540 | C | T | SNP | SYNE1 | NON_SYNONYMOUS_CODING | R/H | cGc/cAc |
| 7 | 44144476 | C | T | SNP | AEBP1 | NON_SYNONYMOUS_CODING | A/V | gCg/gTg |
| 7 | 44150567 | C | T | SNP | AEBP1-1 | NON_SYNONYMOUS_CODING | P/L | cCg/cTg |
| 7 | 50135861 | C | A | SNP | C7orf72 | STOP_GAINED | C/* | tgC/tgA |
| 7 | 63721228 | G | T | SNP | ZNF679 | NON_SYNONYMOUS_CODING | K/N | aaG/aaT |
| 7 | 70800701 | C | T | SNP | WBSCR17 | NON_SYNONYMOUS_CODING | P/L | cCg/cTg |
| 7 | 133948719 | C | T | SNP | LRGUK | NON_SYNONYMOUS_CODING | R/C | Cgc/Tgc |
| 8 | 26371901 | T | A | SNP | DPYSL2 | NON_SYNONYMOUS_CODING | F/L | ttT/ttA |
| 8 | 69243519 | C | T | SNP | C8orf34 | NON_SYNONYMOUS_CODING | P/L | cCg/cTg |
| 8 | 87242173 | C | T | SNP | SLC7A13 | NON_SYNONYMOUS_CODING | A/T | Gct/Act |
| 8 | 89086900 | C | T | SNP | MMP16 | STOP_GAINED | W/* | tgG/tgA |
| 8 | 133984055 | C | T | SNP | TG | STOP_GAINED | R/* | Cga/Tga |
| 8 | 144671428 | C | T | SNP | EEF1D | NON_SYNONYMOUS_CODING | R/H | cGc/cAc |
| 8 | 19811831 | * | -G | DEL | LPL | FRAME_SHIFT | - | - |
| 9 | 104385599 | C | T | SNP | GRIN3A | SPLICE_SITE_DONOR | - | - |
| 9 | 114691873 | G | T | SNP | UGCG | NON_SYNONYMOUS_CODING | D/Y | Gat/Tat |
| 9 | 114874065 | A | G | SNP | SUSD1 | NON_SYNONYMOUS_CODING | V/A | gTc/gCc |
| 9 | 131669658 | G | T | SNP | LRRC8A | NON_SYNONYMOUS_CODING | W/L | tGg/tTg |
| 9 | 139726258 | G | A | SNP | C9orf86 | NON_SYNONYMOUS_CODING | E/K | Gag/Aag |
| 10 | 25684847 | C | A | SNP | GPR158 | NON_SYNONYMOUS_CODING | P/Q | cCa/cAa |
| 10 | 27822783 | G | A | SNP | RAB18 | SPLICE_SITE_DONOR | - | - |
| 10 | 50121830 | C | T | SNP | LRRC18 | NON_SYNONYMOUS_CODING | R/H | cGc/cAc |
| 10 | 55996601 | G | T | SNP | PCDH15 | NON_SYNONYMOUS_CODING | L/I | Ctc/Atc |
| 10 | 89717629 | * | -C | DEL | PTEN | FRAME_SHIFT | - | - |
| 11 | 4674207 | G | T | SNP | OR51E1 | NON_SYNONYMOUS_CODING | A/S | Gct/Tct |
| 11 | 31329285 | G | T | SNP | DCDC1 | STOP_GAINED | S/* | tCa/tAa |
| 11 | 35641259 | C | A | SNP | FJX1 | NON_SYNONYMOUS_CODING | P/T | Ccg/Acg |
| 11 | 47599391 | C | T | SNP | KBTBD4 | NON_SYNONYMOUS_CODING | R/Q | cGg/cAg |
| 11 | 47611638 | G | A | SNP | C1QTNF4 | NON_SYNONYMOUS_CODING | T/M | aCg/aTg |
| 11 | 56086506 | G | T | SNP | OR8K3 | NON_SYNONYMOUS_CODING | A/S | Gcc/Tcc |
| 11 | 59610594 | C | T | SNP | GIF | NON_SYNONYMOUS_CODING | G/S | Ggc/Agc |
| 11 | 62284355 | G | A | SNP | AHNAK | NON_SYNONYMOUS_CODING | T/I | aCa/aTa |
| 11 | 64054488 | T | C | SNP | GPR137 | SPLICE_SITE_DONOR | - | - |
| 11 | 64507531 | G | A | SNP | RASGRP2 | NON_SYNONYMOUS_CODING | A/V | gCg/gTg |
| 11 | 65306697 | C | T | SNP | LTBP3 | NON_SYNONYMOUS_CODING | D/N | Gac/Aac |
| 11 | 78369282 | G | A | SNP | ODZ4 | NON_SYNONYMOUS_CODING | R/W | Cgg/Tgg |
| 11 | 84027872 | G | T | SNP | DLG2 | NON_SYNONYMOUS_CODING | A/E | gCa/gAa |
| 11 | 89911219 | G | A | SNP | NAALAD2 | NON_SYNONYMOUS_CODING | A/T | Gca/Aca |
| 11 | 108183205 | G | T | SNP | ATM | STOP_GAINED | E/* | Gaa/Taa |
| 11 | 134253926 | G | A | SNP | B3GAT1 | NON_SYNONYMOUS_CODING | T/M | aCg/aTg |
| 12 | 7170243 | G | A | SNP | C1S | NON_SYNONYMOUS_CODING | S/N | aGt/aAt |
| 12 | 8926348 | C | A | SNP | RIMKLB | NON_SYNONYMOUS_CODING | L/I | Cta/Ata |
| 12 | 10006979 | C | T | SNP | CLEC2B | SPLICE_SITE_DONOR | - | - |
| 12 | 10584718 | C | G | SNP | KLRC2 | NON_SYNONYMOUS_CODING | A/P | Gct/Cct |
| 12 | 23757430 | G | T | SNP | SOX5 | NON_SYNONYMOUS_CODING | S/Y | tCt/tAt |
| 12 | 81471925 | G | A | SNP | ACSS3 | NON_SYNONYMOUS_CODING | R/H | cGt/cAt |
| 13 | 109792740 | G | A | SNP | MYO16 | NON_SYNONYMOUS_CODING | G/R | Ggg/Agg |
| 14 | 24737588 | C | T | SNP | RABGGTA | NON_SYNONYMOUS_CODING | E/K | Gag/Aag |
| 14 | 53513595 | G | A | SNP | DDHD1 | NON_SYNONYMOUS_CODING | T/M | aCg/aTg |
| 14 | 61446530 | G | T | SNP | TRMT5 | STOP_GAINED | S/* | tCg/tAg |
| 14 | 92361292 | A | T | SNP | FBLN5 | SPLICE_SITE_DONOR | - | - |
| 14 | 94700906 | C | T | SNP | PPP4R4 | STOP_GAINED | R/* | Cga/Tga |
| 14 | 99182629 | G | T | SNP | C14orf177 | NON_SYNONYMOUS_CODING | S/I | aGc/aTc |
| 15 | 35086973 | C | T | SNP | ACTC1 | NON_SYNONYMOUS_CODING | D/N | Gac/Aac |
| 15 | 42052588 | C | T | SNP | MGA | NON_SYNONYMOUS_CODING | A/V | gCg/gTg |
| 15 | 56207510 | C | A | SNP | NEDD4 | NON_SYNONYMOUS_CODING | C/F | tGc/tTc |
| 15 | 83527863 | C | T | SNP | HOMER2 | NON_SYNONYMOUS_CODING | D/N | Gat/Aat |
| 16 | 4432662 | C | A | SNP | VASN | NON_SYNONYMOUS_CODING | A/E | gCa/gAa |
| 16 | 10837916 | G | A | SNP | NUBP1 | NON_SYNONYMOUS_CODING | D/N | Gac/Aac |
| 16 | 20043437 | C | T | SNP | GPR139 | NON_SYNONYMOUS_CODING | A/T | Gcc/Acc |
| 16 | 30566613 | C | T | SNP | ZNF764 | NON_SYNONYMOUS_CODING | A/T | Gcc/Acc |
| 16 | 30780576 | G | A | SNP | RNF40 | NON_SYNONYMOUS_CODING | G/R | Ggg/Agg |
| 16 | 31089392 | C | T | SNP | ZNF646 | NON_SYNONYMOUS_CODING | L/F | Ctc/Ttc |
| 17 | 18024284 | C | A | SNP | MYO15A | NON_SYNONYMOUS_CODING | P/T | Ccc/Acc |
| 17 | 66352939 | G | A | SNP | ARSG | NON_SYNONYMOUS_CODING | R/H | cGt/cAt |
| 17 | 27382890 | * | +TGG | INS | PIPOX | CODON_INSERTION | - | - |
| 18 | 44174396 | G | T | SNP | LOXHD1 | NON_SYNONYMOUS_CODING | Q/K | Cag/Aag |
| 18 | 72913951 | G | A | SNP | ZADH2 | NON_SYNONYMOUS_CODING | T/M | aCg/aTg |
| 19 | 5212481 | C | T | SNP | PTPRS | NON_SYNONYMOUS_CODING | E/K | Gag/Aag |
| 19 | 6177252 | G | A | SNP | ACSBG2 | NON_SYNONYMOUS_CODING | A/T | Gca/Aca |
| 19 | 9060417 | G | T | SNP | MUC16-1 | NON_SYNONYMOUS_CODING | S/Y | tCc/tAc |
| 19 | 9072386 | G | T | SNP | MUC16-2 | NON_SYNONYMOUS_CODING | S/R | agC/agA |
| 19 | 9491853 | G | T | SNP | ZNF559-ZNF177 | NON_SYNONYMOUS_CODING | M/I | atG/atT |
| 19 | 12155657 | G | T | SNP | ZNF878 | NON_SYNONYMOUS_CODING | R/S | Cgt/Agt |
| 19 | 21606080 | G | T | SNP | ZNF493 | STOP_GAINED | E/* | Gag/Tag |
| 19 | 44470693 | T | A | SNP | ZNF221 | NON_SYNONYMOUS_CODING | F/I | Ttt/Att |
| 19 | 54496423 | G | A | SNP | CACNG6 | NON_SYNONYMOUS_CODING | V/M | Gtg/Atg |
| 20 | 55840775 | C | A | SNP | BMP7 | NON_SYNONYMOUS_CODING | S/I | aGc/aTc |
| 20 | 57429700 | C | T | SNP | GNAS | NON_SYNONYMOUS_CODING | T/M | aCg/aTg |
| 20 | 62124537 | G | A | SNP | EEF1A2 | NON_SYNONYMOUS_CODING | T/M | aCg/aTg |
| 22 | 41566474 | C | T | SNP | EP300 | NON_SYNONYMOUS_CODING | H/Y | Cat/Tat |
| 22 | 51043086 | C | T | SNP | MAPK8IP2 | NON_SYNONYMOUS_CODING | P/L | cCg/cTg |
| 22 | 31679151 | * | -CA | DEL | PIK3IP1 | FRAME_SHIFT | - | - |
| X | 140926189 | G | T | SNP | MAGEC3 | NON_SYNONYMOUS_CODING | A/S | Gcc/Tcc |
| X | 153033298 | G | A | SNP | PLXNB3 | NON_SYNONYMOUS_CODING | R/Q | cGg/cAg |

**Patient 2**

| Chr | Position | Wildtype | Mutant | Change type | Gene name | Effect | AA Old/New | Condon Old/New |
| --- | --- | --- | --- | --- | --- | --- | --- | --- |
| 1 | 15809805 | G | A | SNP | CELA2B | NON_SYNONYMOUS_CODING | D/N | Gac/Aac |
| 1 | 22178310 | C | G | SNP | HSPG2 | NON_SYNONYMOUS_CODING | G/A | gGg/gCg |
| 1 | 36384759 | G | T | SNP | EIF2C1 | NON_SYNONYMOUS_CODING | R/L | cGa/cTa |
| 1 | 62228715 | G | A | SNP | INADL | NON_SYNONYMOUS_CODING | R/H | cGc/cAc |
| 1 | 108160190 | C | A | SNP | VAV3 | NON_SYNONYMOUS_CODING | C/F | tGt/tTt |
| 1 | 120437072 | G | T | SNP | ADAM30 | NON_SYNONYMOUS_CODING | Q/K | Cag/Aag |
| 1 | 154938866 | C | A | SNP | SHC1 | STOP_GAINED | G/* | Gga/Tga |
| 1 | 161145865 | C | T | SNP | B4GALT3 | SPLICE_SITE_ACCEPTOR | - | - |
| 2 | 30379554 | C | T | SNP | YPEL5 | NON_SYNONYMOUS_CODING | R/C | Cgt/Tgt |
| 2 | 74689426 | C | T | SNP | MOGS | NON_SYNONYMOUS_CODING | R/Q | cGg/cAg |
| 2 | 135745292 | G | A | SNP | YSK4 | NON_SYNONYMOUS_CODING | P/S | Cca/Tca |
| 2 | 178494202 | C | T | SNP | PDE11A | NON_SYNONYMOUS_CODING | R/Q | cGa/cAa |
| 2 | 179436184 | C | A | SNP | TTN | NON_SYNONYMOUS_CODING | S/I | aGt/aTt |
| 2 | 187627270 | A | C | SNP | FAM171B | NON_SYNONYMOUS_CODING | K/T | aAg/aCg |
| 2 | 210685335 | G | T | SNP | UNC80 | NON_SYNONYMOUS_CODING | G/C | Ggt/Tgt |
| 2 | 211521336 | A | G | SNP | CPS1 | NON_SYNONYMOUS_CODING | S/G | Agc/Ggc |
| 2 | 241570108 | G | A | SNP | GPR35 | NON_SYNONYMOUS_CODING | A/T | Gcc/Acc |
| 2 | 242815023 | C | A | SNP | CXXC11 | NON_SYNONYMOUS_CODING | T/N | aCt/aAt |
| 3 | 436537 | T | C | SNP | CHL1 | NON_SYNONYMOUS_CODING | S/P | Tcc/Ccc |
| 3 | 4693862 | G | A | SNP | ITPR1 | NON_SYNONYMOUS_CODING | R/H | cGt/cAt |
| 3 | 19924104 | T | A | SNP | EFHB | NON_SYNONYMOUS_CODING | T/S | Acc/Tcc |
| 3 | 49318188 | C | G | SNP | USP4 | NON_SYNONYMOUS_CODING | G/A | gGg/gCg |
| 3 | 111426941 | G | A | SNP | PLCXD2 | NON_SYNONYMOUS_CODING | R/H | cGc/cAc |
| 3 | 130313177 | G | A | SNP | COL6A6 | NON_SYNONYMOUS_CODING | R/Q | cGa/cAa |
| 3 | 155199857 | G | C | SNP | PLCH1 | NON_SYNONYMOUS_CODING | L/V | Ctg/Gtg |
| 4 | 62758511 | C | A | SNP | LPHN3 | NON_SYNONYMOUS_CODING | P/T | Cca/Aca |
| 4 | 88533885 | G | T | SNP | DSPP | STOP_GAINED | G/* | Gga/Tga |
| 4 | 126367699 | G | A | SNP | FAT4 | SPLICE_SITE_DONOR | - | - |
| 4 | 162307473 | A | G | SNP | FSTL5 | NON_SYNONYMOUS_CODING | L/S | tTg/tCg |
| 5 | 41181597 | T | A | SNP | C6 | NON_SYNONYMOUS_CODING | N/I | aAt/aTt |
| 5 | 74518160 | G | A | SNP | ANKRD31 | NON_SYNONYMOUS_CODING | L/F | Ctt/Ttt |
| 5 | 112151261 | C | T | SNP | APC | STOP_GAINED | R/* | Cga/Tga |
| 5 | 150275830 | G | A | SNP | ZNF300 | NON_SYNONYMOUS_CODING | P/L | cCt/cTt |
| 6 | 3850779 | C | T | SNP | FAM50B | NON_SYNONYMOUS_CODING | P/L | cCg/cTg |
| 6 | 7888955 | C | T | SNP | TXNDC5 | NON_SYNONYMOUS_CODING | E/K | Gag/Aag |
| 6 | 12122381 | T | C | SNP | HIVEP1 | NON_SYNONYMOUS_CODING | S/P | Tca/Cca |
| 6 | 30917171 | G | T | SNP | DPCR1 | NON_SYNONYMOUS_CODING | R/S | agG/agT |
| 6 | 32609935 | A | T | SNP | HLA-DQA1 | NON_SYNONYMOUS_CODING | K/M | aAg/aTg |
| 6 | 38750892 | G | C | SNP | DNAH8 | NON_SYNONYMOUS_CODING | R/T | aGa/aCa |
| 6 | 152770673 | C | T | SNP | SYNE1 | NON_SYNONYMOUS_CODING | V/I | Gtt/Att |
| 7 | 6737018 | C | T | SNP | ZNF12 | NON_SYNONYMOUS_CODING | E/K | Gaa/Aaa |
| 7 | 31848659 | G | A | SNP | PDE1C | NON_SYNONYMOUS_CODING | S/L | tCa/tTa |
| 7 | 50070741 | C | T | SNP | ZPBP | NON_SYNONYMOUS_CODING | R/H | cGc/cAc |
| 7 | 53103786 | T | C | SNP | POM121L12 | NON_SYNONYMOUS_CODING | I/T | aTc/aCc |
| 7 | 72849407 | A | G | SNP | FZD9 | NON_SYNONYMOUS_CODING | Y/C | tAt/tGt |
| 7 | 92763288 | T | A | SNP | SAMD9L | NON_SYNONYMOUS_CODING | E/V | gAg/gTg |
| 7 | 92763289 | C | A | SNP | SAMD9L | STOP_GAINED | E/* | Gag/Tag |
| 7 | 122635132 | A | C | SNP | TAS2R16 | NON_SYNONYMOUS_CODING | V/G | gTt/gGt |
| 7 | 124386977 | G | A | SNP | GPR37 | NON_SYNONYMOUS_CODING | R/W | Cgg/Tgg |
| 7 | 131191353 | C | A | SNP | PODXL | STOP_GAINED | E/* | Gaa/Taa |
| 7 | 131853201 | C | T | SNP | PLXNA4 | NON_SYNONYMOUS_CODING | R/H | cGt/cAt |
| 7 | 136700016 | G | A | SNP | CHRM2 | NON_SYNONYMOUS_CODING | R/Q | cGg/cAg |
| 7 | 150873332 | G | A | SNP | ASB10 | NON_SYNONYMOUS_CODING | S/L | tCg/tTg |
| 8 | 1497427 | C | A | SNP | DLGAP2 | NON_SYNONYMOUS_CODING | H/N | Cac/Aac |
| 8 | 24193068 | G | A | SNP | ADAM28 | NON_SYNONYMOUS_CODING | G/D | gGc/gAc |
| 8 | 31498155 | G | A | SNP | NRG1 | NON_SYNONYMOUS_CODING | A/T | Gcg/Acg |
| 8 | 35093382 | C | T | SNP | UNC5D | NON_SYNONYMOUS_CODING | A/V | gCg/gTg |
| 8 | 52321392 | T | A | SNP | PXDNL | NON_SYNONYMOUS_CODING | K/M | aAg/aTg |
| 8 | 77766530 | T | G | SNP | ZFHX4 | NON_SYNONYMOUS_CODING | L/R | cTt/cGt |
| 8 | 104898169 | T | G | SNP | RIMS2 | NON_SYNONYMOUS_CODING | L/V | Ttg/Gtg |
| 9 | 27949805 | C | T | SNP | LINGO2 | NON_SYNONYMOUS_CODING | E/K | Gaa/Aaa |
| 9 | 35799675 | C | T | SNP | NPR2 | NON_SYNONYMOUS_CODING | R/C | Cgt/Tgt |
| 9 | 118949523 | G | A | SNP | PAPPA | NON_SYNONYMOUS_CODING | R/H | cGc/cAc |
| 10 | 26508182 | C | T | SNP | GAD2 | NON_SYNONYMOUS_CODING | T/I | aCt/aTt |
| 10 | 35929166 | C | T | SNP | FZD8 | NON_SYNONYMOUS_CODING | V/M | Gtg/Atg |
| 10 | 45920489 | G | A | SNP | ALOX5 | NON_SYNONYMOUS_CODING | R/H | cGc/cAc |
| 10 | 115391684 | C | T | SNP | NRAP | NON_SYNONYMOUS_CODING | G/R | Gga/Aga |
| 10 | 115889716 | C | T | SNP | C10orf118 | NON_SYNONYMOUS_CODING | D/N | Gat/Aat |
| 11 | 3735057 | C | T | SNP | NUP98 | STOP_GAINED | W/* | tgG/tgA |
| 11 | 24518806 | C | T | SNP | LUZP2 | NON_SYNONYMOUS_CODING | A/V | gCg/gTg |
| 11 | 48347007 | T | A | SNP | OR4C3 | NON_SYNONYMOUS_CODING | V/E | gTg/gAg |
| 11 | 55322161 | T | C | SNP | OR4C15 | NON_SYNONYMOUS_CODING | S/P | Tca/Cca |
| 11 | 62363306 | C | A | SNP | MTA2 | NON_SYNONYMOUS_CODING | C/F | tGt/tTt |
| 11 | 117376186 | C | T | SNP | DSCAML1 | NON_SYNONYMOUS_CODING | R/H | cGc/cAc |
| 11 | 124134859 | A | T | SNP | OR8G5 | NON_SYNONYMOUS_CODING | K/M | aAg/aTg |
| 11 | 130275737 | C | T | SNP | ADAMTS8 | NON_SYNONYMOUS_CODING | E/K | Gag/Aag |
| 11 | 133795862 | T | C | SNP | IGSF9B | SPLICE_SITE_ACCEPTOR | - | - |
| 12 | 9001336 | C | A | SNP | A2ML1 | NON_SYNONYMOUS_CODING | F/L | ttC/ttA |
| 12 | 10339143 | G | A | SNP | C12orf59 | NON_SYNONYMOUS_CODING | V/I | Gtc/Atc |
| 12 | 48258938 | G | C | SNP | VDR | NON_SYNONYMOUS_CODING | L/V | Cta/Gta |
| 12 | 51389498 | T | G | SNP | SLC11A2 | NON_SYNONYMOUS_CODING | I/L | Att/Ctt |
| 12 | 53454736 | C | T | SNP | TENC1 | NON_SYNONYMOUS_CODING | P/S | Ccc/Tcc |
| 13 | 20315782 | C | T | SNP | PSPC1 | NON_SYNONYMOUS_CODING | M/I | atG/atA |
| 13 | 36744849 | A | G | SNP | CCDC169-SOHLH2 | NON_SYNONYMOUS_CODING | L/P | cTg/cCg |
| 13 | 103490998 | C | A | SNP | BIVM-ERCC5 | NON_SYNONYMOUS_CODING | A/E | gCa/gAa |
| 13 | 111371782 | G | T | SNP | ING1 | NON_SYNONYMOUS_CODING | A/S | Gcg/Tcg |
| 16 | 1675995 | C | T | SNP | CRAMP1L | NON_SYNONYMOUS_CODING | S/L | tCg/tTg |
| 16 | 15711247 | C | T | SNP | KIAA0430 | NON_SYNONYMOUS_CODING | G/S | Ggc/Agc |
| 16 | 23646651 | C | T | SNP | PALB2 | NON_SYNONYMOUS_CODING | A/T | Gca/Aca |
| 16 | 50750801 | G | T | SNP | NOD2 | SPLICE_SITE_ACCEPTOR | - | - |
| 16 | 51174374 | G | A | SNP | SALL1 | NON_SYNONYMOUS_CODING | P/S | Ccc/Tcc |
| 16 | 58562402 | C | A | SNP | CNOT1 | NON_SYNONYMOUS_CODING | D/Y | Gac/Tac |
| 17 | 7577547 | C | T | SNP | TP53 | NON_SYNONYMOUS_CODING | G/D | gGc/gAc |
| 17 | 35937502 | G | A | SNP | SYNRG | STOP_GAINED | Q/* | Caa/Taa |
| 17 | 38975310 | * | -CGCCGTGGCCGC | DEL | KRT10 | CODON_DELETION | - | - |
| 17 | 38975316 | * | -GGCCGCCGCCGT | DEL | KRT10 | CODON_DELETION | - | - |
| 17 | 37871562 | C | T | SNP | ERBB2 | NON_SYNONYMOUS_CODING | P/L | cCg/cTg |
| 17 | 39913922 | G | A | SNP | JUP | NON_SYNONYMOUS_CODING | L/F | Ctc/Ttc |
| 17 | 78367246 | T | A | SNP | RNF213 | NON_SYNONYMOUS_CODING | V/D | gTc/gAc |
| 19 | 16611911 | G | A | SNP | C19orf44 | NON_SYNONYMOUS_CODING | R/Q | cGg/cAg |
| 19 | 35800821 | G | A | SNP | MAG | NON_SYNONYMOUS_CODING | D/N | Gac/Aac |
| 19 | 41709472 | C | T | SNP | CYP2S1 | NON_SYNONYMOUS_CODING | A/V | gCg/gTg |
| 19 | 44793043 | G | C | SNP | ZNF235 | NON_SYNONYMOUS_CODING | S/C | tCt/tGt |
| 19 | 46119788 | G | C | SNP | EML2 | NON_SYNONYMOUS_CODING | H/Q | caC/caG |
| 19 | 54080503 | T | G | SNP | ZNF331 | NON_SYNONYMOUS_CODING | L/R | cTc/cGc |
| 19 | 54407962 | C | T | SNP | PRKCG | NON_SYNONYMOUS_CODING | S/L | tCg/tTg |
| 20 | 17608235 | C | T | SNP | RRBP1 | NON_SYNONYMOUS_CODING | A/T | Gcg/Acg |
| 20 | 25459843 | C | T | SNP | NINL | SPLICE_SITE_ACCEPTOR | - | - |
| 20 | 45131429 | G | T | SNP | ZNF334 | STOP_GAINED | Y/* | taC/taA |
| 20 | 51872391 | G | A | SNP | TSHZ2 | NON_SYNONYMOUS_CODING | M/I | atG/atA |
| 20 | 62038068 | C | T | SNP | KCNQ2 | NON_SYNONYMOUS_CODING | G/R | Ggg/Agg |
| 20 | 62593947 | G | T | SNP | ZNF512B | NON_SYNONYMOUS_CODING | T/N | aCc/aAc |
| 21 | 44513237 | * | +TC | INS | U2AF1 | FRAME_SHIFT | - | - |
| 22 | 39497308 | T | A | SNP | APOBEC3H | NON_SYNONYMOUS_CODING | C/S | Tgc/Agc |
| 22 | 46611073 | C | A | SNP | PPARA | NON_SYNONYMOUS_CODING | T/K | aCg/aAg |
| 22 | 46658705 | C | T | SNP | PKDREJ | NON_SYNONYMOUS_CODING | R/Q | cGg/cAg |
| X | 27998329 | C | T | SNP | DCAF8L1 | NON_SYNONYMOUS_CODING | V/I | Gta/Ata |
| X | 125685736 | A | C | SNP | DCAF12L1 | NON_SYNONYMOUS_CODING | F/V | Ttc/Gtc |
| X | 152721808 | G | A | SNP | HAUS7 | NON_SYNONYMOUS_CODING | A/V | gCc/gTc |

**Table S6. Summary of single cell sampling**

| ID | Samples | Single cells for the region | Tumor cells with SCNAs |
| --- | --- | --- | --- |
| Patient 1 | Region A | 10 | 6 |
|  | Region B | 10 | 5 |
|  | Region C | 10 | 5 |
|  | Region D | 10 | 8 |
|  | Blood | - | - |
| Patient 2 | Region A | 10 | 6 |
|  | Region B | 10 | 9 |
|  | Region C | 10 | 8 |
|  | Region D | 8 | 6 |
|  | Region E | 10 | 6 |
|  | Blood | - | - |
